# Author Correction: Guanidine aptamers are present in vertebrate RNAs associated with calcium signaling and neuromuscular function

**DOI:** 10.1038/s41467-025-64474-z

**Published:** 2025-09-24

**Authors:** Kumari Kavita, Aya Narunsky, Jessica J. Mohsen, Isha Mahadeshwar, Michael G. Mohsen, Yu-Shin Chang, Ronald R. Breaker

**Affiliations:** 1 Department of Molecular, Cellular and Developmental Biology, Yale University, New Haven, CT 06511 USA; 2 Department of Chemistry, Yale University, New Haven, CT 06511 USA; 3 Institute for Biomolecular Design and Discovery, Yale University, West Haven, CT 06516 USA; 4 Department of Molecular Biophysics and Biochemistry, Yale University, New Haven, CT 06511 USA

**Keywords:** Biophysics, Riboswitches, RNA

Correction to: *Nature Communications* 10.1038/s41467-025-62815-6, published online 09 August 2025

In the version of this article originally published, legends for Fig. 5a, b, now listing panel a: consensus sequence, b: pie chart, were interchanged and are now amended in the HTML and PDF versions of the article.

