# Supplementary material for: Guanidine aptamers are present in vertebrate RNAs associated with calcium signaling and neuromuscular function
Source: Nat Commun. 2025 Aug 9;16:7362. doi: 10.1038/s41467-025-62815-6 (PMC12335538; doi:10.1038/s41467-025-62815-6)
Supplement: Supplementary file 1 — Supplementary Information [file 41467_2025_62815_MOESM1_ESM.pdf]

Supplementary Information

Guanidine aptamers are present in vertebrate RNAs associated with calcium signaling and neuromuscular function

Kumari Kavita[1], Aya Narunsky[1], Jessica J. Mohsen[2,3], Isha Mahadeshwar[1], Michael G. Mohsen[1], Yu-Shin Chang[1] and Ronald R. Breaker[1,4*]

[1]Department of Molecular, Cellular and Developmental Biology, Yale University, New Haven, Connecticut 06511, USA, [2]Department of Chemistry, Yale University, New Haven, Connecticut 06511, USA, [3]Institute for Biomolecular Design and Discovery, Yale University, West Haven, Connecticut, 06516, USA, [4]Department of Molecular Biophysics and Biochemistry, Yale University, New Haven, Connecticut 06511, USA

***Correspondence** and requests should be addressed to R.R.B.
email: ronald.breaker@yale.edu

```
NC_087355.1 - Loxodonta africana - African bush elephant
                                       Hairpin 1                   Hairpin 2                   Hairpin 3
NC_087355.1/9292075-9292166     AUGACUGGGGAUGACCC-AGAAAGACCGAAAGCUGGGGACGACCCCAGAAAGACCAAAAGCUGGGGACGACCCCAGAAAGACCG *
                                       Hairpin 4                   Hairpin 5                   Hairpin 6
NC_087355.1/9292167-9292250     AAAGCUGGGGAUGACCCCAGAAAGACCGAAAGCUGGGGACGACCCCAGAAAGACCGAAAGCUGGGGACGACCCCAGAAAGACCG *
                                       Hairpin 7                   Hairpin 8                   Hairpin 9
NC_087355.1/9292251-9292333     AAAGCUGGGGACGACCCCAGAAAGACCGAAAGCU-GGGACGACCCUAGAAAGACCGAAAGCUGGGGACGACCCCAGAAAGACCG *

NC_064833.1 - Elephas maximus - Asian elephant
NC_064833.1/83506617-83506526   AUGACUGGGGACGACCC-AGAAAGACCGAAAGCUGGGGACGACCCCAGAAAGACCGAAAGCUGGGGACGACCCCAGAAAGACCG *
NC_064833.1/83506525-83506442   AAAGCUGGGGACGACCCCAGAAAGACCGAAAGCUGGGGACGACCCCAGAAAGACCGAAAGCUGGGGACGACCCCAGAAAGACCG *
NC_064833.1/83506441-83506358   AAAGCUGGGGACGACCCCAGAAAGACCGAAAGCUGGGGACGACCCCAGAAAGACCGAAAGCUGGGGACGACCCCAGAAAGACCG *
NC_064833.1/83506357-83506274   AAAGCUGGGGACGACCCCAGAAAGACCGAAAGCUGGGGACGACCCUAGAAAGACCGAAAGCUGGGGACGACCCCAGAAAGACCG *

NC_022031.1 - Microtus ochrogaster - Prairie vole (rodent)

NC_022031.1/58512039-58511945   -GAAGGCUGGACGGCCUGGAUUAGGUGGCACAGCACAUGUGGGAUAUGUGGGAUUCUUCUCUGUCUGCACAGGAGCAAAGGACGGUCUAGCAGAUU **

NW_026902293.1 - Neopsephotus bourkii - Bourke's parrot (bird)
NW_026915724.1 - Pezoporus occidentalis - Night parrot (bird)

NW_026902293.1/3626704-3626770  GAGGAAGUUGACGACGCCUUCU--ACAGGCAGCUGAGAACGGCCUCACAGUCACAGGCCG *
NW_026915724.1/7355224-7355158  GAGGAAGUUGACGAGGCCUUCU--ACAGGCAGCUGAGAGCGGCCUCACAGUCACAGGCCG *

NC_086285.1 - Chroicocephalus ridibundus - Black-headed gull (bird)

NC_086285.1/128943152-128943052 CGACACCGGGACGGCCACCGGCUGCCGGGCCCGCUCGCCGGCCGCGCCCCCUGCCCCUGCCCCGCCGGGGGACGACACCCGCCGGCGGGGGCGG **

NC_084596.1 - Cololabis saira - Pacific saury (fish)

NC_084596.1/21215360-21215293   GUCACGGAGGAGGGGGUGCCCGACCGUUCCCGCUGGUCGGCCCGUCGCCGAGGACGGCCACCCGACCG                                    *
NC_084596.1/21215284-21215178   GUCGCCGAGGACGGCCACCC-GACCGUUCCCGCUGGUCGGCCCGUCGCCGAGGACGGCCGCCCGACGGGGCUCGGCGGUGGUCUGUACCAGGAAGGCCC *

NW_023397406.1 - Fundulus heteroclitus - Mummichog (fish)

NW_023397406.1/603764-603851    GCUCCGGCCGAACAGGAAAGUCUGGUGCGGGCGUCGUGGAGGACGACCCCGGCACGACG--AACUAGAAAUAGGAGUAGCAUCAACUAAG **
NW_023397406.1/603674-603761    GCUCCGGCCGGACAGGAAAGUCAGAGGCGGGCGCCGUUGUGGACGGCCCC--GACGAAGCAAAGUUGAGCGGCCCGGAGUCCGCAGUCUU **
NW_023397406.1/603584-603671    GCUCCGGCCGAGCAGGAAAGUCAGAGGCGGUCGCCGUGGUGGGCGGCCCC--GACGAGGCAAAGUCGAGCGGCCUGGAGUCCACAGUCUG **
NW_023397406.1/603494-603581    GUUCCGGCCGAACAGAAAAGUCAAAAGCGGGCGCCGUGGUGGACGGCCCC--GACGAGGCAAAGACGAGCGGCCCGGAGUCCGCAGUCUG **
NW_023397406.1/603404-603491    GCUCCGGCCGAACAGAAAAGUCAAAGGCGGGCGCCGUGGUGGACGGCCCC--GACGAGGCAAAGUCAAGCGGCCUGGAGACCGCGGUCGG **

* = Antisense to CA8 Intron
** = CA8 Intron
```

Supplementary Fig. 1 (previous page) │ Guanidine-II aptamer consensus hits associated with vertebrate *CA8* genes. Predicted base-paired stems are highlighted with blue, green, or yellow shading. Red nucleotides correspond to the highly conserved GGACGRCC consensus sequences of the P1 and P2 loops. *Notes: Loxodonta africana* (nine hairpins depicted) and *Elephas maximus* (12 hairpins depicted) appear to have emerged by relatively recent duplications due to the similarity of each hairpin and linker region. This repetitive character is also apparent in the hits in fish, again suggesting a relatively recent duplication in evolutionary history to create tandem hairpin arrangements. Such similarity is not apparent in some other guanidine-II aptamer candidates in other vertebrate species. Exceptions include rare instances of hairpin duplication in the *CACNA1C* gene (Supplementary Fig. 11). Hairpin 1 of *Loxodonta africana* carries a natural C-to-U nucleotide change relative to the bacterial consensus model. It is known that a single identical nucleotide change to bacterial constructs disrupts guanidine binding[3]. However, this mutant hairpin appears to support binding of guanidine by hairpin 2 (Supplementary Fig. 6, Supplementary Fig. 7).

Name	Description	Diseases	Hits
Unknown	Commonly caused by lack of annotations of the resident genome		68
PISD	Phosphatidylserine Decarboxylase, Phosphatidylethanolamine synthesis	Liberfarb Syndrome, Fundus Dystrophy	9
CACNA1C	Ca^{2+} Transporter	Long QT Syndrome	2
DAZAP2	DAZ Associated Protein 2, binds CALCOCO2 (Ca^{2+} binding protein)	Azoospermia, Visual Epilepsy	2
GRIA1	Glutamate Ionotropic Receptor 1, Transports Na^{+} or Ca^{2+}	Intellectual Developmental Disorder, Autism	2
CARD11	Caspase Recruitment Domain Family Member 11	Immune System Dysfunction	2
SRFBP1	Serum Response Factor-Binding Protein, regulates SRF	Aneurysm, Cutis Laxa (Ca^{2+} deposits)	1
CRTC1	CREB-regulated Transcription Coactivator 1, Ca^{2+} regulated	Various Carcinomas	1
SMTN	Smoothelin	Spondylometaphyseal Dysplasia, etc...	1
FBXL14	F-box and Leucine-rich Repeat Protein 14		1
PTPRK-like	Receptor-type Tyrosine-protein Phosphatase KAPPA Isoform		1
KIF23	Kinesin Family Member 23, Ca^{2+}-dependent	Various Anemias	1
?	Melanotransferrin-like		1
ZBTB16	Zinc Finger and BTB Domain Containing 16	Skeletal Defects, Leukemia, etc...	1
AKIRIN2	Gene Regulation Factor, Involved in Calcineurin Signaling	Immune System Dysfunction, Dyskinesia	1
TALPID3	KIAA0586, Centrosomal Protein, Involved in Bone Formation	Joubert Syndrome, Short-Rib Dysplasia	1
CA9	Carbonic Anhydrase 9, Similar to CA8	Cancer	1
LRRC4	Leucine-rich Repeat Containing 4, Forms Ca^{2+}-transport Complex	Autism	1
FBXO25	F-box Protein 25	Autism	1
ARHGAP36	Rho GTPase Activating Protein 36, Involved in Bone Formation	Hypotrichosis, Pheochromocytoma	1
EDN3	Endothelin-3, Ca^{2+} Regulation	Waardenburg Synd., Hirschprung Dis. 4	1
ARHGAP29	Rho GTPase Activating Protein 29, Interacts with CACNA1C	Cleft Lip	1
ZNF395	Zinc Finger Protein 395	Huntington's Disease	1
SETD7	SET Domain Containing 7, Relevant to Ca^{2+} Signaling	Cancer	1
PLEKHB2	Phosphatidylinositol-3,4,5-triphosphate binding	Kallman Syndrome, Articulation Disorder	1
?	Phosphatidylinositol 4,5-bisphosphate 3-kinase (subunit alpha)		1
TNFRSF8		Prostate Cancer	1
NOS1	Nitric Oxide Synthase 1, Ca^{2+} activated	Idiopathic Achalasia, Pyloric Stenosis	1
FBX039		Ophthalmia Neonatorum	1
RHOT1	Ras Homolog Family Member T1, Ca^{2+} responsive	Parkinson's Disease	1
SNX27	Sorting Nexin 27, Phosphatidylinositol-binding	Charcot-Marie-Tooth Disease	1
SCARA5	Scavenger Receptor Class A, Member 5, Ca^{2+} binding	Osteogenesis Imperfecta, Galloway-Mowat	1
SCFD2	Sec1 Family Domain-Containing Protein 2	Multiple Personality Disorder	1

Supplementary Fig. 2 | List of gene associations for most guanidine-II riboswitch candidates present in the Rfam database. Unknown indicates that the genome annotations presently available via NCBI do not reveal an associated gene. Gene descriptions or diseases with strong relevance to Ca^{2+} are highlighted in light blue.

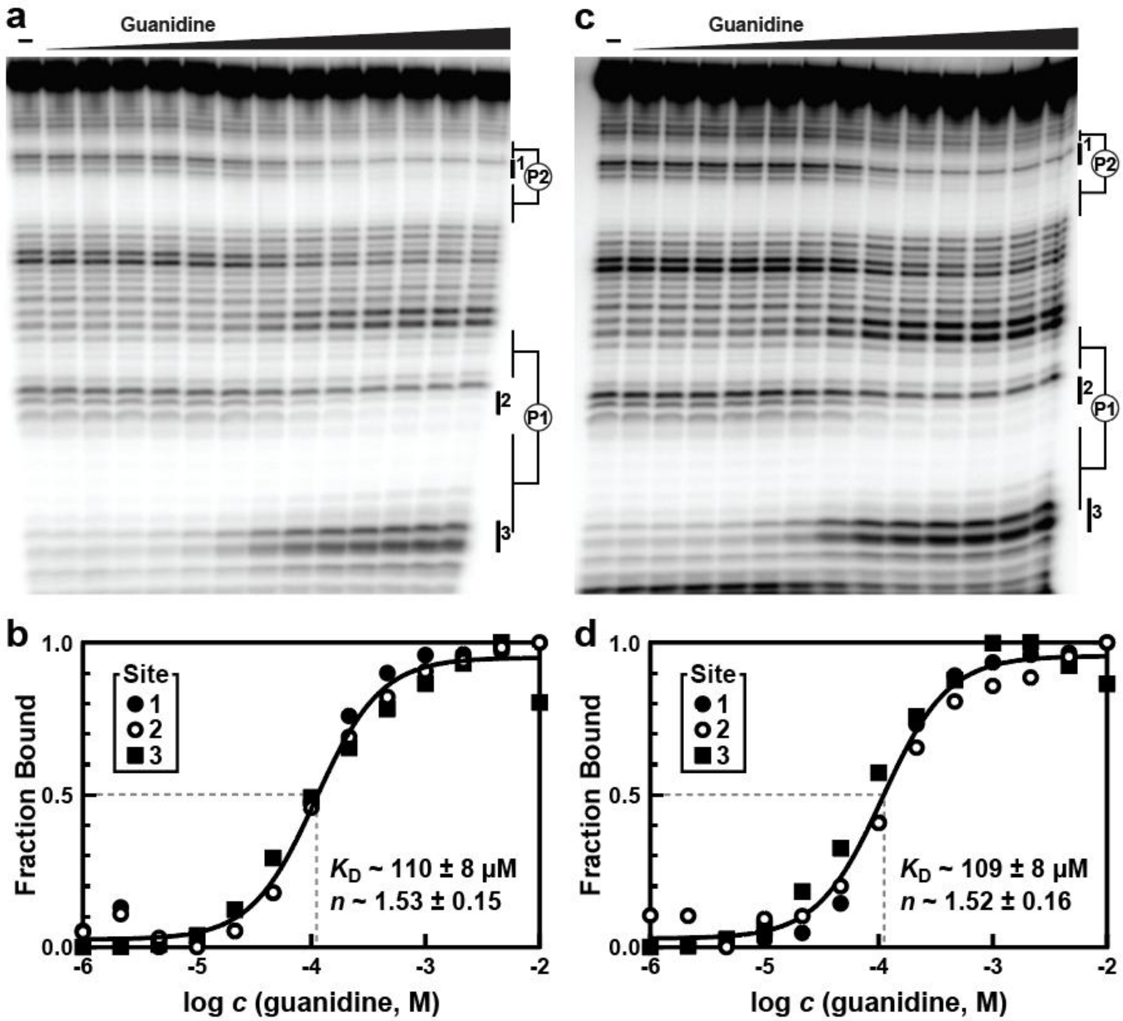

Supplementary Fig. 3 │ Replicates of in-line probing analysis of the *CA8* elephant aptamer using the ligand guanidine. a Autoradiogram depicting PAGE separation of in-line probing reaction products containing trace amounts of 5′ ^{32}P-labeled *CA8* elephant aptamer RNA construct (Fig. 2a) incubated in the absence of ligand (–), or with increasing concentrations of guanidine, ranging from 10^{-6} M to 10^{-2} M at third-log intervals. The reactions were as conducted as described for **Fig. 2**. NR, T1, and $^{-}$OH indicate precursor RNAs subjected to no reaction, partial digestion with RNase T1 (cleaves after G nucleotides), and partial digestion at elevated pH, respectively. Pre indicates the band corresponding to the precursor (full-length) 5′ ^{32}P-labeled RNA. Other annotations are as described for Fig. 2b. **b** Plot of the logarithm of guanidine concentration vs the fraction of RNA bound as determined by band intensity analysis of the autoradiogram depicted in a. The error values are the standard error of the mean determined by goodness of fit to a sigmoidal curve. **c**, **d** A third representative of the in-line probing analysis. Annotations are as described for a and b.

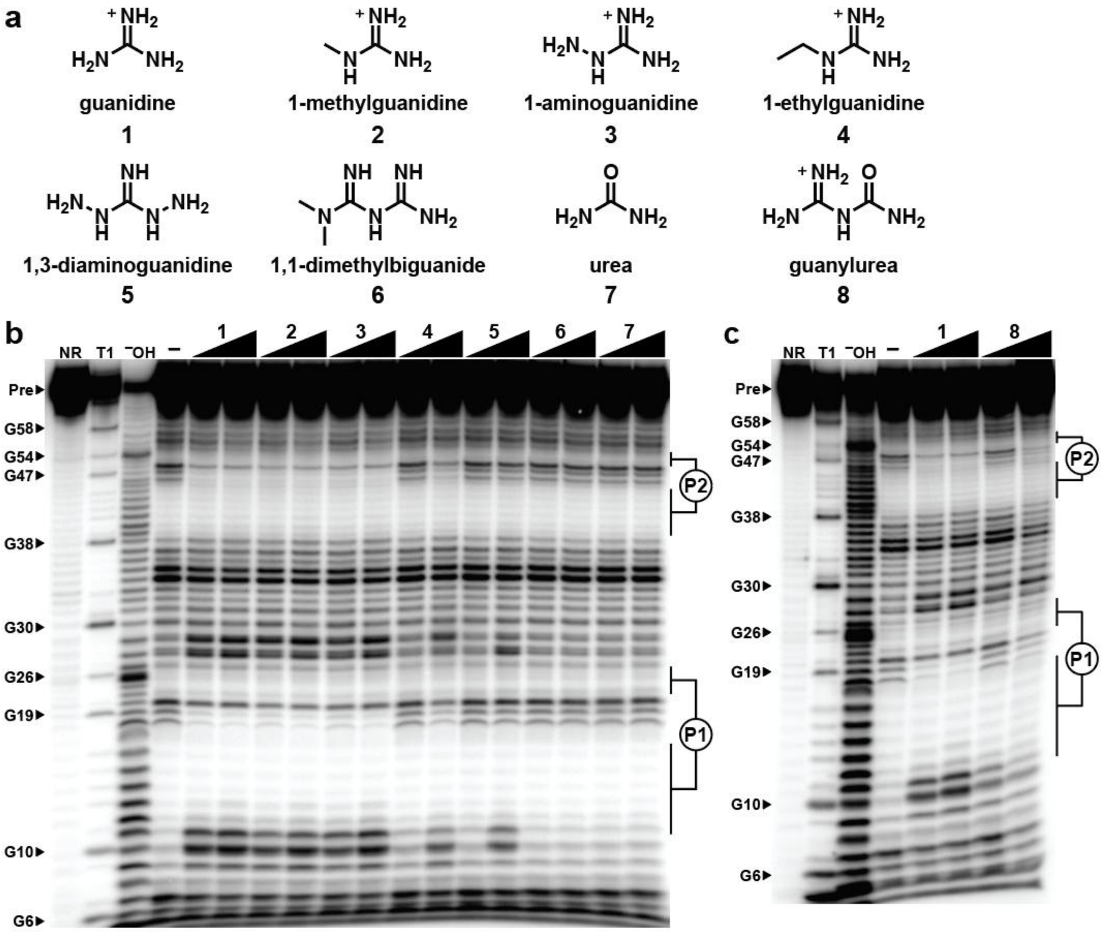

Supplementary Fig. 4 │ Selectivity of ligand binding by the elephant *CA8* guanidine-II aptamer. a Chemical structures of guanidine (in its protonated guanidinium form) and other guanidine-related compounds used to determine the structure-activity relationship for the aptamer. **b** Autoradiogram depicting PAGE separation of in-line probing reaction products derived from the 5′ ^{32}P-labeled *CA8* elephant aptamer RNA construct (Fig. 2a) incubated in the absence of a candidate ligand (–) or with compounds **1** through **7** (at 1 or 10 mM from left to right). Annotations are as described for Fig. 2b. **c** Results of in-line probing analyses using compounds **1** or **8**. Annotations are as described for b. Although the final designs of many experiments were based on the findings from scout experiments, the assays were not repeated under identical conditions and therefore conclusions from the data in Supplementary Fig. 4 are based only on the assays depicted (n = 1).

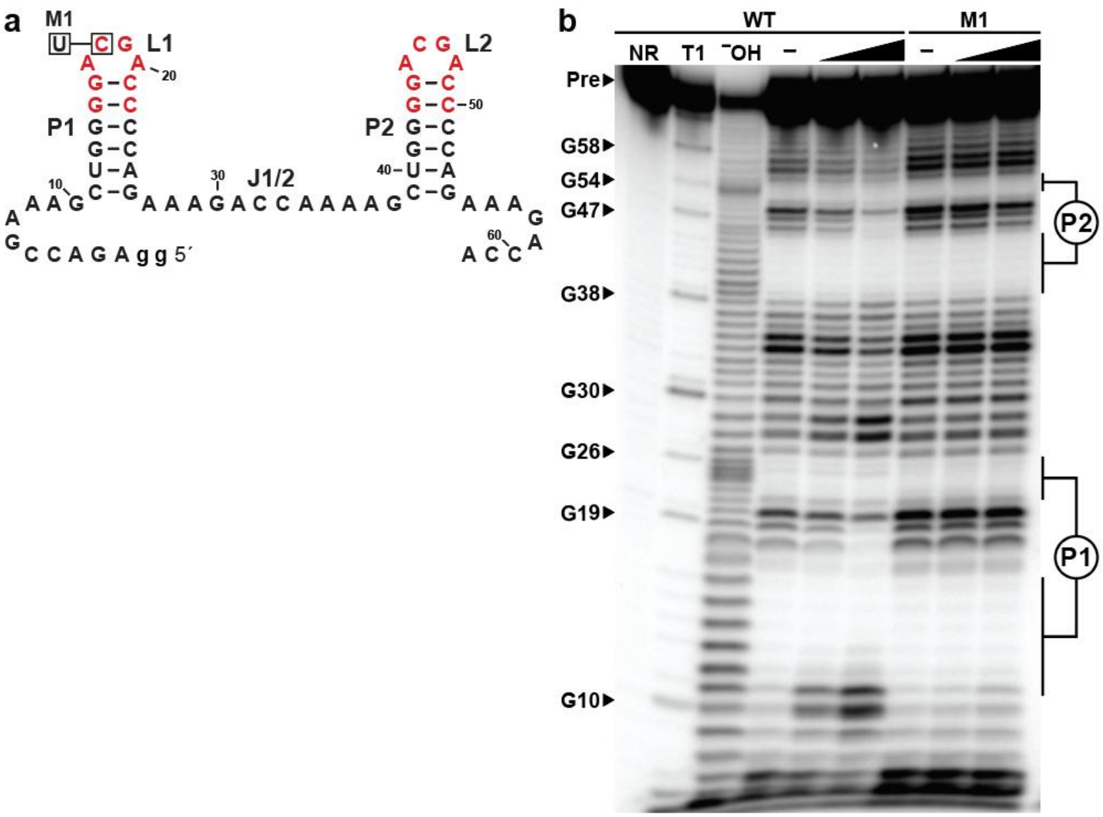

Supplementary Fig. 5 │ Mutation to a conserved nucleotide in L1 causes a loss of guanidine affinity. a Sequence and secondary structure model of the *CA8* elephant aptamer RNA construct (**Fig. 2**), depicting the highly conserved nucleotides of guanidine-II aptamers (red) and a C18U mutation (construct M1) known in bacterial riboswitch representatives to disrupt ligand binding[3]. **b** Autoradiogram depicting PAGE separation of in-line probing reaction products from 5′ ^{32}P-labeled WT and M1 RNAs incubated in the absence of ligand (–) or with guanidine (100 µM or 1 mM from left to right). NR, T1, and ⁻OH indicate precursor RNAs subjected to no reaction, partial digestion with RNase T1 (cleaves after G nucleotides), and partial digestion at elevated pH, respectively. Pre indicates the band corresponding to the precursor (full-length) 5′ ^{32}P-labeled RNA. Other annotations are as described for Fig. 2b. Although the final designs of many experiments were based on the findings from scout experiments, the assays were not repeated under identical conditions and therefore conclusions from the data in Supplementary Fig. 5 are based only on the assays depicted (n = 1).

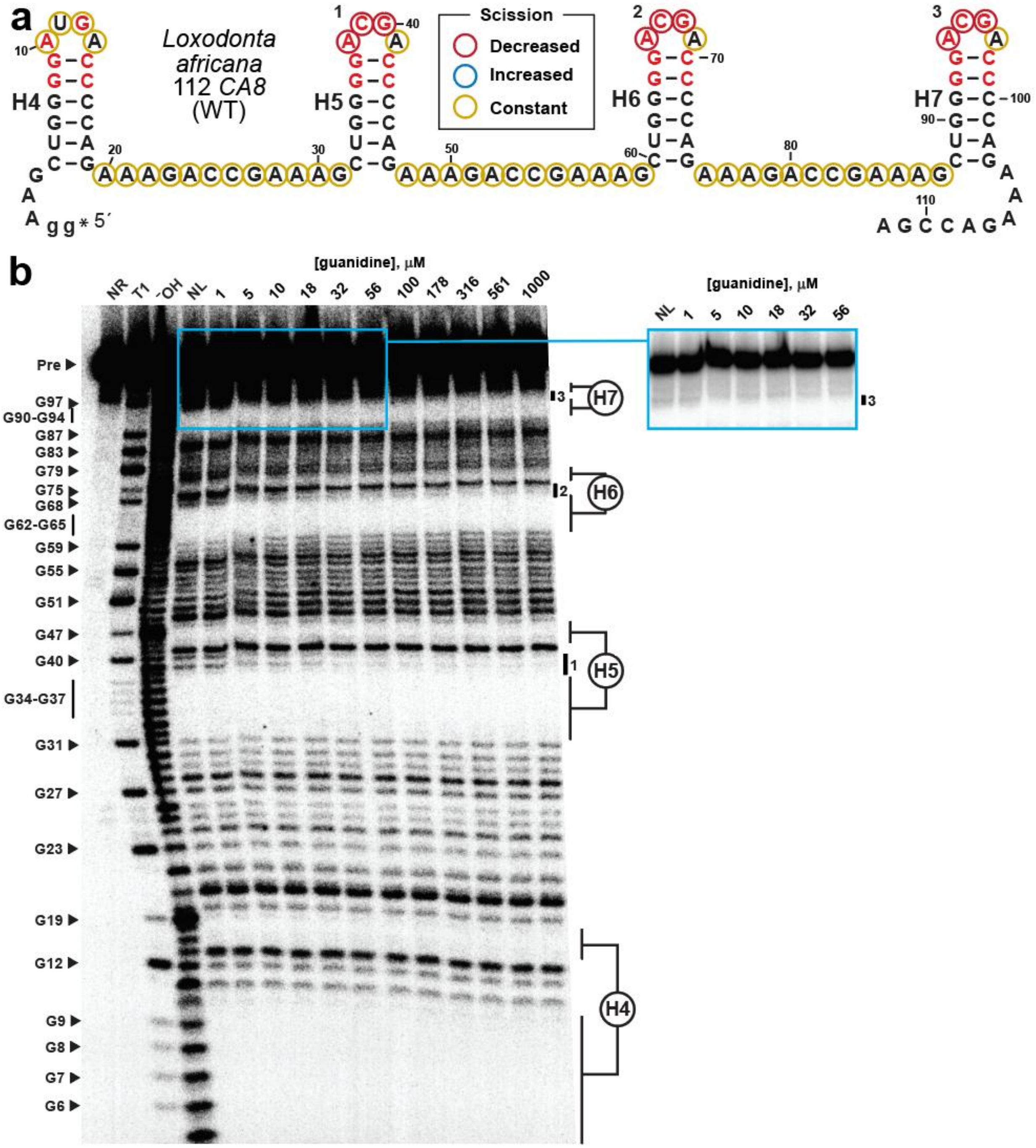
a
Loxodonta africana 112 CA8 (WT)
Scission
Decreased
Increased
Constant
H4
H5
H6
H7
b
[guanidine], µM
NR
T1
⁻OH
NL
1
5
10
18
32
56
100
178
316
561
1000
Pre
G97
G90-G94
G87
G83
G79
G75
G68
G62-G65
G59
G55
G51
G47
G40
G34-G37
G31
G27
G23
G19
G12
G9
G8
G7
G6

Supplementary Fig. 6 (Previous Page) │ Guanidine binding by an RNA construct containing four hairpin structures from the *CA8* gene region of African elephant. a Sequence and secondary structure model of a WT 112 *CA8* RNA construct carrying 112 nucleotides encompassing hairpins H4 through H7 of the nine predicted hairpins similar to those observed for guanidine-II riboswitch aptamers in the antisense orientation of the *Loxodonta africana CA8* gene. The lowercase g letters identify guanosine nucleotides added to the construct to promote production by in vitro transcription. Nucleotides are numbered beginning with position 1 as the first natural nucleotide of the RNA synthesized. Annotations, including the sites of band intensity modulation, are derived from the in-line probing assay depicted in b. **b** Representative in-line probing analysis of the 112-nucleotide RNA construct depicted in a. The inset presents a lower intensity image of the autoradiogram region identified with the blue box to illustrate the change in band intensity corresponding to the loop of H7 (at or near nucleotides 94-96 of the construct). Sites 1, 2 and 3 (vertical bars) identify bands that undergo intensity modulation upon guanidine binding, corresponding to binding by H5, H6, and H7, respectively. Additional annotations are as described for Fig. 2. Note that the apparent K_D for this RNA (estimated from this representative data set to be no poorer than ~5 μM) is better than that observed for the analogous M1 construct (Supplementary Fig. 7, Supplementary Figure 8). Although the final designs of many experiments were based on the findings from scout experiments, the assays were not repeated under identical conditions and therefore conclusions from the data in Supplementary Fig. 6 are based only on the assays depicted (n = 1).

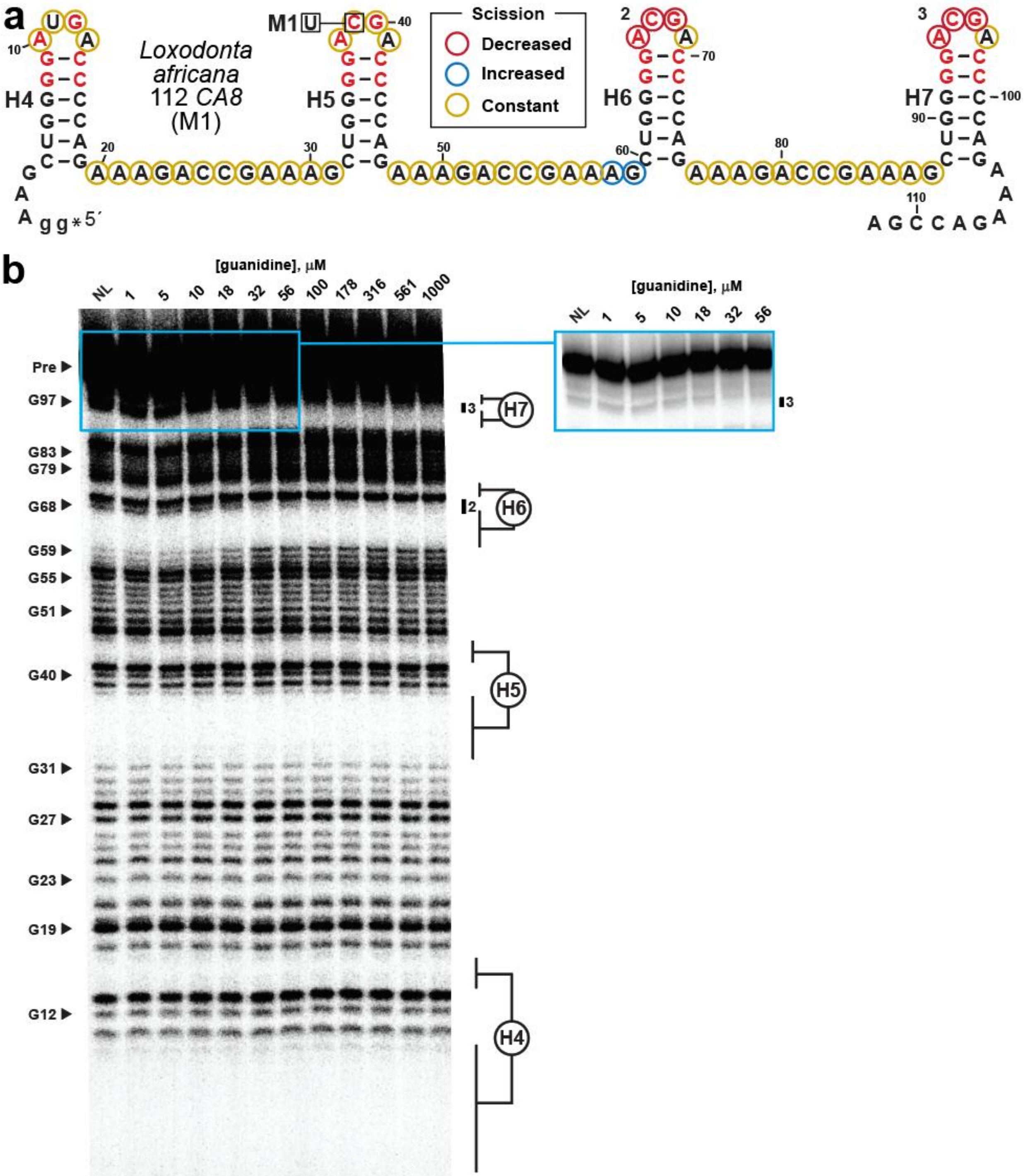
a
Loxodonta africana
112 CA8
(M1)
H4
H5
H6
H7
M1
Scission
Decreased
Increased
Constant
b
[guanidine], µM
NL 1 5 10 18 32 56 100 178 316 561 1000
NL 1 5 10 18 32 56
Pre
G97
G83
G79
G68
G59
G55
G51
G40
G31
G27
G23
G19
G12

Supplementary Fig. 7 (Previous Page) │ Guanidine binding by the M1 mutant construct derived from the WT 112 RNA construct containing four hairpin structures from the *CA8* gene region of African elephant. a Sequence and secondary structure model of a mutant version of the 112 *CA8* RNA construct encompassing hairpins H4 through H7 from an antisense transcript of the *Loxodonta africana CA8* gene. This M1 mutant carries a C to U mutation at position 39 in the loop of H5. Other annotations are as described for Supplementary Fig. 6a. **b** Representative in-line probing analysis of the 112 *CA8* M1 RNA construct depicted in a. Other annotations are as described for Supplementary Fig. 6b. Note the loss of band intensity modulation by guanidine in the loop of H5. Inset: an image of the boxed area of the in-line probing autoradiogram depicted with lower intensity reveals modulation of band intensity at site 3. Note that sites of modulation in this representative in-line probing analysis all undergo half maximal modulation between data points generated with 18 and 32 μM guanidine, indicating that the apparent K_D for this RNA is better than that observed for the construct carrying only H2 and H3 (Fig. 2). Although the final designs of many experiments were based on the findings from scout experiments, the assays were not repeated under identical conditions and therefore conclusions from the data in Supplementary Fig. 7 are based only on the assays depicted (n = 1).

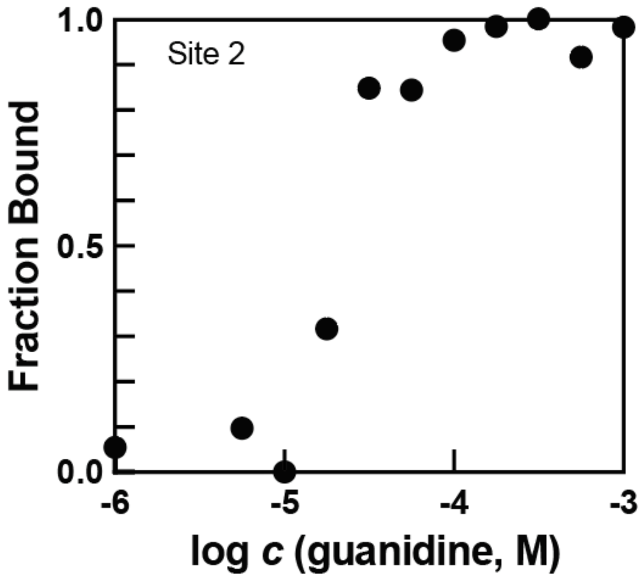

Supplementary Fig. 8 │ Plot of the fraction of M1 RNA bound to ligand versus the logarithm of the molar concentration of guanidine. Data are based on the in-line probing assay for the 112-nucleotide M1 (Supplementary Fig. 7) RNA construct derived from African elephant (see Fig. 2c for additional details). The K_D for guanidine binding to H6 and H7 is estimated from this single representative in-line probing assay to be ~20 µM for this M1 version of the 112 *CA8* RNA construct based on the *Loxodonta Africana* representative.

PISD Hits: Rfam

```
LR584245.2/12918029-12917966     GACUACGUCUGUCCCGGGACGGCCUGGGACUACGUCU------GUCCCGGGACGGCCUGGGACUACGUCU
VXBO01001565.1/237799-237868     CACCACAUCACCUCAGGGACGGCCCUGAGCUCACACCACAUCACCUCAGGGACGGCCCUGAGCUCACACC
VWPO01000134.1/570213-570282     CACCACAUCACCUCAGGGACGACCCUGUCCCCACACCACAUCACCUCAGGGACGGCCCUGUCCC
VXAT01001751.1/404196-404127     CAUCACAUCGCCUCAGGGACGGCCCUGUCCCCCCACCACAUCCCCUCAGGGACGGCCCUGUCCCCCCACC
VZTS01007580.1/59979-59910       CACCACAUCACCUCAGGGACGGCCCUGUCCCCCCACCACAUUGCUGCAGGGACGGCCCUGUCCCCAGACC
QCWP01025089.1/245-313           ACAGCCAACCCCAUAGGGACGACCCUAUAGGGACAGCCAAC-CCCAUAGGGACGGCCCCAUAGGGAGCCC
VYZM01023727.1/19269-19338       CAUCAGGUCCCCUCGGGGACGGCCCUGAGCCCACAGCACGUCCCCUCGGGGACGGCCCUGUCCCCCUCCC
VXBH01000815.1/12831-12900       CAUCGGGUCCCCUCAGGGACGGCCCUGAGCCCACAUCGGGCCCCCUCAGGGACGGCCCUAUCGUUCCCCC
VXBO01001565.1/237941-238005     CAUCACGUCCCCUCAGGGACGACCCUAAUCCCAAAUCAC-----CUCAGGGACGACCCUGAGCCCACAUC
VZRW01003191.1/625580-625649     UGUCACCUUCCCUCAGGGACGGCCCUGUCCCCGCACCACCCCGCCUCAGGGACGGCCCUGUCCCCACAGC
```

```
    LR584245.2: Takifugu rubripes (fish)
VXBO01001565.1: Rhinoptilus africanus (Double-banded courser) (bird)
VWPO01000134.1: Glareola pratincole (Collared pratincole) (bird)
VXAT01001751.1: Mesembrinibis cayennensis (Green ibis) (bird)
VZTS01007580.1: Pluvianellus socialis (Magellanic plover) (bird)
QCWP01025089.1: Phasianus colchicus (Ring-necked Pheasant) (bird)
VYZM01023727.1: Dromas ardeola (Crab-plover) (bird)
VXBH01000815.1: Rynchops niger (Black skimmer) (bird)
VZRW01003191.1: Aegotheles bennettii (Barred owlet-nightjar) (bird)
```

Supplementary Fig. 9 │ Alignment of guanidine-II aptamer candidates located ~10 kb upstream of the *PISD* coding region.

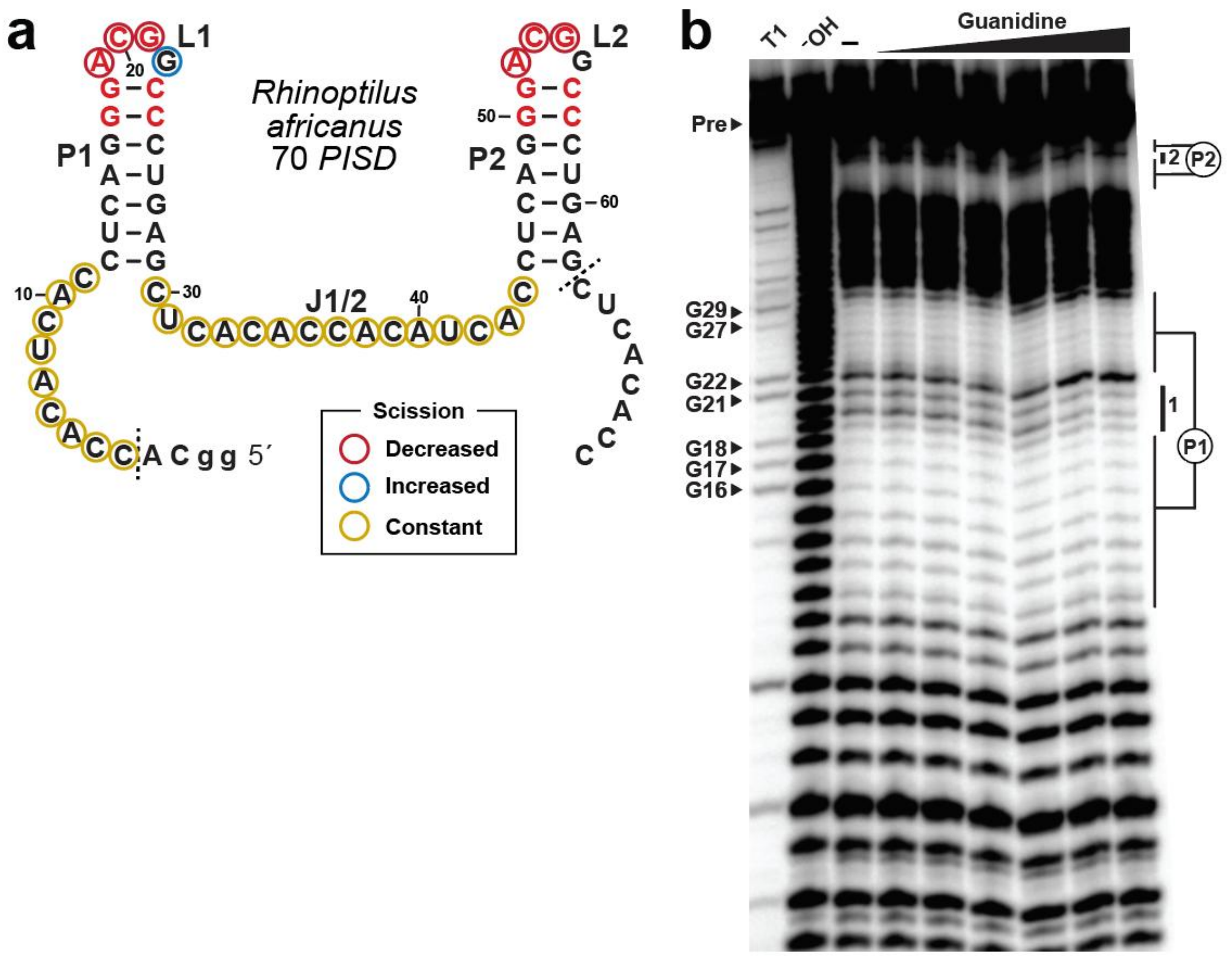

Supplementary Fig. 10 │ Ligand binding by the guanidine-II aptamer candidate associated with the *Rhinoptilus africanus PISD* gene. a Sequence and secondary structure for the *Rhinoptilus africanus* RNA construct (called 70 *PISD*) encompassing 70 nucleotides from a location ~10 kb upstream of the *PISD* mRNA. Annotations are as described for Fig. 2a, with locations of strand scission characteristics approximated downstream of the J1/2 region based on the data in b. **b** Image of the polyacrylamide gel electrophoresis (PAGE) separation of the products of representative in-line probing reactions using the 70 *PISD* RNA construct from *Rhinoptilus africanus*. Reactions are conducted in the absence of guanidine (–) and with the following, ascending concentrations as indicated: 1 nM, 1 µM, 10 µM, 100 µM, 1 mM, and 10 mM. Annotations are as described for Fig. 2b. Although the final designs of many experiments were based on the findings from scout experiments, the assays were not repeated under identical conditions and therefore conclusions from the data in Supplementary Fig. 10 are based only on the assays depicted (n = 1).

```
Hairpin Candidates                                P1                                 P1 Duplicate                                 P2                           Atypical
NC_047044.1/100992796-100992912  GAGCCCACAGGACGGCCUGUGGAAGCAUCAGCUCUCCCC--------------------------------GCCGGCCU-GUCCUGCACGAGCAGGGACGAGAAAGCAGCAGGA-ACGGA-UCCAGCCCCCGUAA
NC_064569.1/1774520-1774404      GAGCCCACAGGACGGCCUGUGGAAGCAUCAGCUCUCCCC--------------------------------GCCGGCCU-GUCCUGCACGAGCAGGGACGAGAAAGCAGCAGGA-ACGGA-UCCAGCCCCCGUAA
NC_083105.1/102785261-102785377  GAGCCCACAGGACGGCCUGUGUGAGCGUCAGCUCUCCC--------------------------------UGCCGGUCU-GUCCUGCACGAGCAGGGACGAGAAAGCAGCAGGA-ACGGA-UCCAGCCCCCGUAA
NW_020838027.1/1162953-1162837   GAGCCCACAGGACGGCCUGUGUGAGCGUCAGCUCUCCCC--------------------------------ACUGGCCU-GUCCUGCACGAGCAGGGACGAGAAAGCAGCAGGA-ACGGA-UCCAGCCCCUGUAA
NC_082693.1/2027474-2027372      GAGCCCACAGGACGGCCUGUGGAAGCAUCAGCUCUCCCC--------------------------------GCCGGCCU-GUCCUGCACGAGCAGGGACGACAAAGCAGCAGGA-ACGGA-UCCAGCCCCCGUAA
NC_082648.1/1172417-1172300      GAGCCCACAGGACGGCCUGUGUAAGCGUCCGCUCUCCCG-------------------------------CUCCGGCUC-GUCCCGCACAAACAGGGAGGAGGAAGCAGCAGGA-ACGGA-GCCAGCCCCCGUGA
NC_090836.1/1528406-1528289      GAGCCCACAGGACGGCCUGUGUAAGCGUCAGCUCUCCCG-------------------------------CUCCGGCUC-GUCCCGCACAAACAGGGAGGAGAAAGCAGCAGGA-ACGGA-GCCAGCCCCCCGUG
NC_080074.1/106035088-106035205  GAGCCCACAGGACGGCCUGUGUAAGCGUCCGCUCUCCCG-------------------------------CUCCGGCUC-GUCCUGCGCAAACAGGGAGGAGGAAGCAGCAGGG-ACGGA-GCCAGCCCCCGUGA
NC_083726.1/115948631-115948748  GAGCCCACAGGACGGCCUGUGUAAGCGUCAGCUCUCCCG-------------------------------CUCCGGCUU-GUCCUGCACAAACAGGGAGGAGAAAGCAGCAGCA-ACGGA-GCCAGCCCCCGUGA
NC_082671.1/3151204-3151087      GAGCCCACAGGAGGGCCUGUGUAAGCGCCAGCUCUCCCG-------------------------------CGCCGGCCU-GUCCUGCACAAACAGGGAGGAGAAAGCAGCAGGA-ACGGA-ACCGGCCCCUGUAA
NC_045794.1/1192106-1191989      GAGCCCACAGGACGGCCUGCGUAAGCGUCCGCUCUCCCG-------------------------------CUCCGGCUC-GUCCCGCACAAACAGGGAGGAGAAAGCAGCAGGA-ACGGA-GCCAGCCCCCGUGA
NC_081321.1/1205072-1204955      GAGCCCACAGGACGGCCUGCGUACGCGUCAGCCCUCCCC-------------------------------CUCCGGCUC-GCCCUGCACAAACACAGAGGAGAAAGCAGCGGGG-ACGGAG-CCAGCCCCCGUAA
NC_080197.1/80764760-80764877    AUCUCCACAGGAUGGCCUGCGUAAGCAUCAGCUCUCCAG-------------------------------CUCCAGCUC-AUCCUGCACGAACAGGGAGGCCAGAGCAGCAUGG-ACGGAA-CCAGCUCCUGUAA
NW_020176571.1/1134497-1134380   GAGCCCACAGGAUGGCCUGUGUAAGCGUCAGCCCUCCCG-------------------------------CGCCGGCCG-GUCCUGCGCAAGCAGGGAGGAGAAAGCAGCA-GGAACGGA-UCCAGCCCCCGUAA
NC_041219.1/53723190-53723073    GAGCCCACAGGAUGGCCUGGGUAAGCGUCAGCUCUCCCC-------------------------------CUCCGGCUU-GCCCCGCACAAACACGGAGGAGAAAGCAGCCGGA-ACGGA-GCCAGCCCCUGUAA
NW_006797390.1/8535772-8535890   GAGCCCACAGGAUGGCCUGUGUAAGCGUCAGCUCGCCCG-------------------------------CGCCGGCCU-GUCCUGCACAAACAGGGAGGAGAAAGCAGCAGGA-ACGGA-GCCAGCCCCCGUAA
NW_021703783.1/1181510-1181393   GAGCCCACAGGAUGGCCUGUGUAAGCGUCAGCUCUCCCG-------------------------------UGCCGGCCU-GUCCUGCACAAGCAGGGAGGAGAAAGCAGCAGGA-ACGGA-UCCAGCCCCCGUAA
NW_022098015.1/1219300-1219183   GAGCCCACAGGAUGGCCUGUGUAAGCGUCAGCUCUCCCG-------------------------------UGCCGGCCU-GUCCUGCACAAGCAGGGAGGAGAAAGCAGCAGGA-ACGGA-UCCAGCCCCCGUAA
NC_089229.1/101008963-101009080  GAGCCCACAGGAUGGCCUGUGUAAGCGUCAGCCCUCCCG-------------------------------CGCCGGCCG-GUCCUGCGCAAGCAGGGAGGAGAAAGCAGCAGGA-ACGGA-UCCAGCCCCCGUAA
NC_045772.1/101634598-101634715  GAGCCCACAGGAUGGCCUGUGUAAGCGUCAGCCCUCCCG-------------------------------CGCCGGCCG-GUCCUGCGCAAGCAGGGAGGAGAAAGCAGCAGGA-ACGGA-UCCAGCCCCCGUAA
NC_010447.5/69148213-69148325    GAUGCCAUAGGGUGGCCUGUGUACCUGUCUGCCCUCU-------------------------------------GGCCGCGUCGUGCACAAAGCAGGGAGAACAGAGGGCCGGCAACGG--GUGGGUUCCCCUAC
NC_090306.1/1389022-1388873      GAGCCCACAGGACGGCCUGUGUGAGCGUCAGCCCAGCCCACAGGACGGCCUGUGUGAGCGUCAGCUCUCCCCGCUGGCCUGUCCUGCACGAGCAGGGACGAGAAAGCAGCAGGA-AUGGA-UCCAGCCCCUGUAA
NC_083323.1/101274366-101274515  GAGCCCACAGGACGGCCUGUGUGAGCGUCAGCCCAGCCCACAGGACGGCCUGUGUGAGCGUCAGCUCUCCCCGCUGGCCUGUCCUGCACGAGCAGGGCCGAGAAAGCAGCAGGA-AUGGA-UCCAGCCCCUGUAA
```

NC_047044.1 – *Tursiops truncates* – Common bottlenose dolphin
NC_064569.1 – *Orcinus orca* – Orca
NC_083105.1 – *Lagenorhynchus albirostris* – White-beaked dolphin
NW_020838027.1 – *Lagenorhynchus obliquidens* – Pacific white-sided dolphin
NC_082693.1 – *Delphinus delphis* – Short-beaked common dolphin
NC_082648.1 – *Balaenoptera ricei* – Rice's whale
NC_090836.1 – *Eschrichtius robustus* – Gray whale
NC_080074.1 – *Balaenoptera acutorostrata* – Common minke whale
NC_083726.1 – *Eubalaena glacialis* – North Atlantic right whale
NC_082671.1 – *Mesoplodon densirostris* – Blainville's beaked whale
NC_045794.1 – *Balaenoptera musculus* – Blue whale
NC_081321.1 – *Kogia breviceps* – Pygmy sperm whale
NC_080197.1 – *Hippopotamus amphibius* – Hippopotamus
NW_020176571.1 – *Neophocaena asiaeorientalis* – Narrow-ridged finless porpoise
NC_041219.1 – *Physeter catodon* – Sperm whale
NW_006797390.1 – *Lipotes vexillifer* – Baiji
NW_021703783.1 – *Monodon monoceros* – Narwhal
NW_022098015.1 – *Delphinapterus leucas* – Beluga whale
NC_089229.1 – *Phocoena phocoena* – Harbour porpoise
NC_045772.1 – *Phocoena sinus* – Vaquita
NC_010447.5 – *Sus scrofa* – Wild boar
NC_090306.1 – *Pseudorca crassidens* – False killer whale
NC_083323.1 – *Globicephala melas* – Long-finned pilot whale

```
Additional Hairpin Candidates
NC_047044.1/100992937-100993015  GAGCCCACAGGACGGCCUGUGGAAGCGUCAGCUGAGCCCACAGGACGGCCUGUGUGAGCGUC
NC_064569.1/1774379-1774301      GAGCCCACAGGACGGCCUGUGGAAGCGUCAGCUGAGCCCACAGGACGGCCUGUGUGAGCGUC
```

NC_047044.1 – *Tursiops truncates* – Common bottlenose dolphin
NC_064569.1 – *Orcinus orca* – Orca

Supplementary Fig. 11 (previous page) │ Guanidine-II aptamer consensus hits associated with vertebrate *CACNA1C* genes, which code for calcium voltage-gated channel subunit alpha1 C proteins. The sequences depicted are present in the sense orientation within an intron of the *CACNA1C* pre-mRNA. Predicted base-paired stems are highlighted with green (P1-like) or blue (P2-like) shading. Red nucleotides correspond to the highly conserved GGACGRCC consensus sequences of the P1 and P2 loops. *Top*: P1 and P2 hairpin candidates or atypical hairpin candidates of the various species indicated. Note that two species (false killer whale and long-finned pilot whale) exhibit a duplication of P1 (underlined nucleotides), which is similar to that occurring with the CA8 gene of elephant (Supplementary Fig. 1). *Bottom*: Additional candidate hairpins in the *CACNA1C* pre-mRNA of the common bottlenose dolphin and orca located downstream of the other hairpin candidates depicted above.

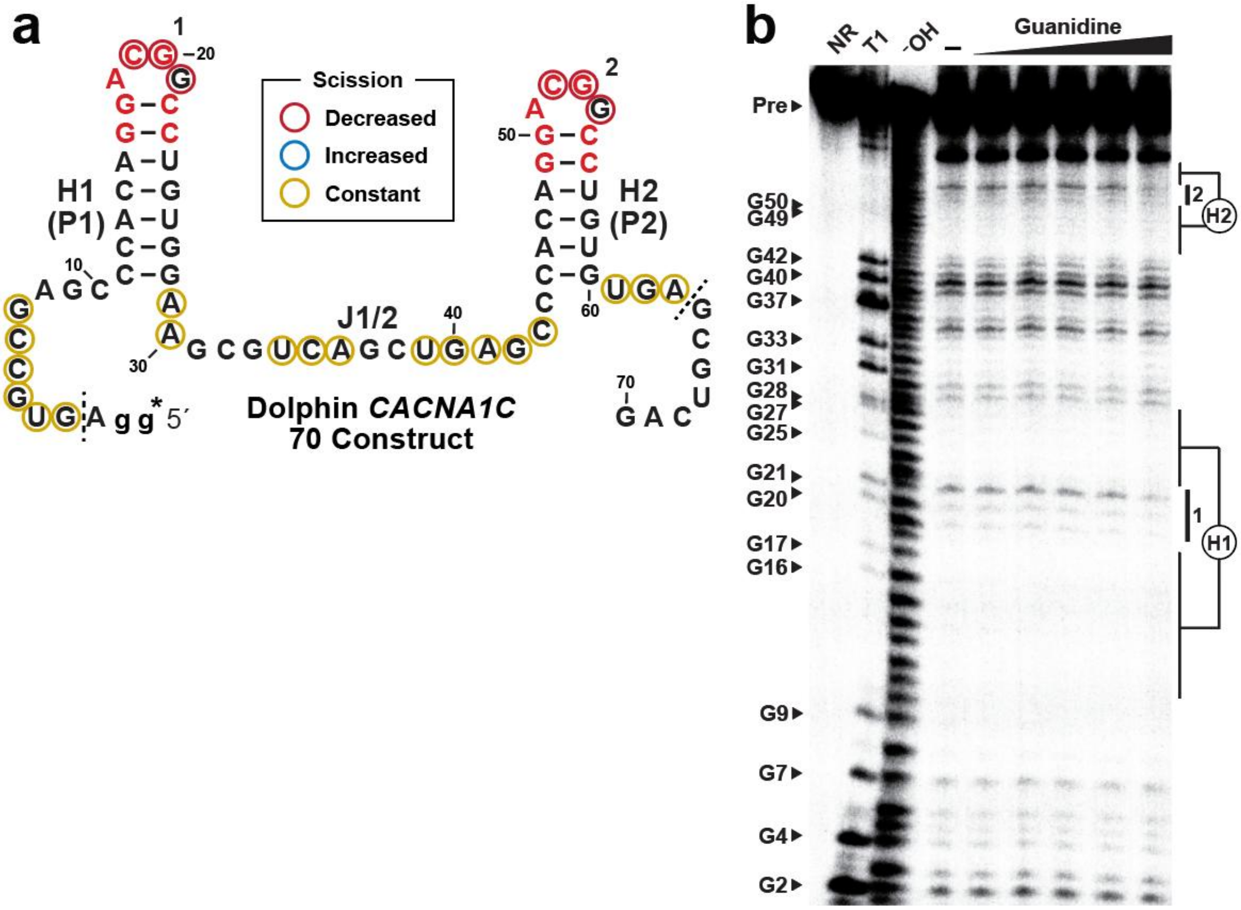

Supplementary Fig. 12 │ In-line probing analysis of a candidate guanidine-II aptamer system from the *CACNAC1* gene of dolphin. a Sequence and secondary structure model of a 70-nucleotide RNA encompassing hairpins H1 and H2 from an antisense transcript of the *Tursiops truncatus CACNA1C* gene. **b** In-line probing analysis of the 70-nucleotide RNA construct depicted in a. Concentrations of guanidine used ranged from 10 µM to 10 mM in 10-fold increments. Annotations are as described for Fig. 2. Although the final designs of many experiments were based on the findings from scout experiments, the assays were not repeated under identical conditions and therefore conclusions from the data in Supplementary Fig. 12 are based only on the assays depicted (n = 1).

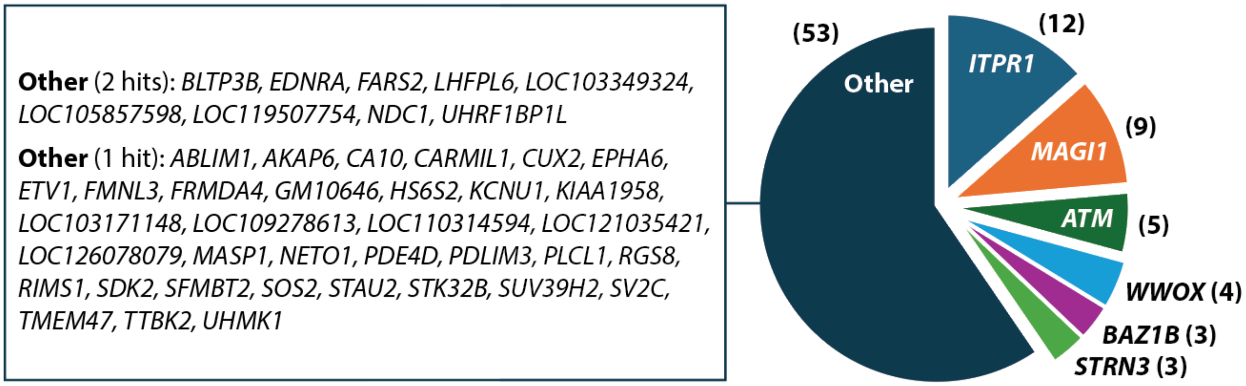

Supplementary Fig. 13 │ Vertebrate genes associated with the initial hits from a search of the bacterial guanidine-I consensus. The number in parentheses indicate the hits for each gene identified in the initial bioinformatic search conducted with an e-value cut-off of 10. See the main text for descriptions of the top-ranked genes and how these are relevant to Ca^{2+} signaling and neuromuscular function.

```
                                                P1                 *    P1′     P2         P2′                       * *
NC_073643.1/23512860-23512741   GGUUUUCUAAAAUUAGUGUUCUCUAGUACAUAAACCAGUAAGAACAGAGUAUUUAAUUAAAUAUUACACAAGGAUAUAAAAGCCCAGGGGAAUGCAGUC
NC_059177.1/21768532-21768413   GGUUUUCUAAAAUUAGUGUUCUCUAGUACAUAAACCAGUAAGAACAGAGUAUUUAAUUAAAUAUUACACAAGGAUAUAAAAGCCCAGGGGAAUGCAGUC
NC_056072.1/21814524-21814405   GGUUUUCUAAAAUUAGUGUUCUCUAGUACAUAAACCAGUAAGAACAGAGUAUUUAAUUAAAUAUUACACAAGGAUAUAAAAGCCCAGGGGAAUGCAGUC
NC_091078.1/67425017-67424898   GGUUUUCUAAAAUUAGUGUUCUCUAGUACAUAAACCAGUAAGAACAGAGUAUUUAAUUAAAUAUUACACAAGGAUAUAAAAGCCCAGGGGAAUGCAGUC
NC_083889.1/21849216-21849097   GGUUUUCUAAAAUUAGUGUUCUCUAGUACAUAAACCAGUAAGAACAGAGUAUUUAAUUAAAUAUUACACAAGGAUAUAAAAGCCCAGGGGAAUGCAGUC
NW_026572372.1/39226704-39226823 GGUUUUCUAAAAUUAGUGUUCUCUAGUACAUAAACCAGUAAGAACAGAGUAUUUAAUUAAAUAUUACACAAGGAUAUAAAAGCCCAGGGGAAUGCAGUC
NW_024070193.1/38509970-38510089 GGUUUUCUAAAAUUAGUGUUCUCUAGUACAUAAACCAGUAAGAACAGAGUAUUUAAUUAAAUAUUACACAAGGAUAUAAAAGCCCAGGGGAAUGCAGUC
NC_032671.1/22507305-22507186   GGUUUUCUAAAAUUAGUGUUCUCUAGUACAUAAACCAGUAAGAACAGAGUAUUUAAUUAAAUAUUACACAAGGAUAUAAAAGCCCAGGGGAAUGCAGUC
NW_011494766.1/1382341-1382460  GGUUUUCUAAAAUUAGUGUUCUCUAGUACAUAAACCAGUAAGAACAGAGUAUUUAAUUAAAUAUUACACAAGGAUAUAAAAGCCCAGGGGAAUGCAGUC
NC_091638.1/22048269-22048150   GGUUUUCUAAAAUUAGUGUUCUCUAGUACAUAAACCAGUAAGAACAGAGUAUUUAAUUAAAUAUUACACAAGGAUAUAAAAGCCCAGGGGAAUGCAGUC
NC_030829.1/21673050-21672931   GGUUUUCUAAAAUUAGUGUUCUCUAGUACAUAAACCAGUAAGAACAGAGUAUUUAAUUAAAUAUUACACAAGGAUAUAAAAGCCCAGGGGAAUGCAGUC
NW_005816350.1/3475122-3475003  GGUUUUCUAAAAUUAGUGUUCUCUAGUACAUAAACCAGUAAGAACAGAGUAUUUAAUUAAAUAUUACACAAGGAUAUAAAAGCCCAGGGGAAUGCAGUC
NC_037349.1/21830857-21830738   GGUUUUCUAAAAUUAGUGUUCUCUAGUACAUAAACCAGUAAGAACAGAGUAUUUAAUUAAAUAUUACACAAGGAUAUAAAAGCCCAGGGGAAUGCAGUC
NC_040097.1/38996850-38996969   GGUUUUCUAAAAUUAGUGUUCUCUAGUACAUAAACCAGUAAGAACAGAGUAUUUAAUUAAAUAUUACACAAGGAUAUAAAAGCCCAGGGGAAUGCAGUC
NC_010455.5/61110940-61111059   GGUUUUCUAAAAUUAGUGUUCUCUAGUACAUAAACCAGUAAGAACAGAGUAUUUAAUUAAAUAUUACACAAGGAUAUAAAAGCCCAGGGGAAUGCAGUC
NC_062544.1/141367516-141367635 GGUUUUCUAAAAUUAGUGUUCUCUAGUACAUAAACCAGUAAGAACAGAGUAUUUAAUUAAAUAUUACACAAGGAUAUAAAAGCCCAGGGGAAUGCAGUC
NC_089252.1/81106421-81106302   GGUUUUCUAAAAUUAGUGUUCUCUAGUACAUAAACCAGUAAGAACAGAGUAUUUAAUUAAAUAUUACACAAGGAUAUAAAAGCCCAGGGGAAUUGCAGU
NC_083704.1/27139085-27138966   GGUUUUCUAAAAUUAGUGUUCUCUAGUACAUAAACCAGUAAGAACAGAGUAUUUAAUUAAAUAUUACACAAGGAUAUAAAAGCCCAGGGGAAUUGCAGU
NC_069699.1/19503506-19503387   GGUUUUCUAAAAUUAGUGUUCUCUAGUACAUAAACCAGUAAGAACAGAGUAUUUAAUUAAAUAUUACACAAGGAUAUAAAAGCCCAGGGGAAUUGCAGU
NW_012134636.1/2638533-2638652  GGUUUUCUAAAAUUAGUGUUCUCUAGUACAUAAACCAGUAGGAACAGAGUAUUUAAUUAAAUAUUAUACAAGGGUAUAAAAGCCCAAGGGAAUUCAGUC
NC_068910.1/38743130-38743249   GGUUUUCUAAAAUUAGUGUUCUCUAGUACAUAAACCAGUAAGAACAGAGUAUUUAAUUAAAUAUUACACAAGAAUAUAAAAGCCCAGGGGAAUGCAGUC
NC_080198.1/11256947-11256828   AGUUUUCUAAAAUUAGUGUUCUCUAGUACAUAAACCAGUAAUAACAGAGUAUUUAAUUAAAUAUUACACAAGGAUAUAAAAGCCCAGGGGAAUGUAGUC
NC_057838.1/33968950-33968831   GGUUUUCUAAAAUUAGCGUUCUCUAGUACAUAAACCAGUAAGAACAGAGUAUUUAAUUAAAUAUUACACAAGGAUAUAAAAGCCCAGGGGAAUUGCAGU
NC_057407.1/37683297-37683416   GGUUUUCUAAAAUUAGCGUUCUCUAGUACAUAAACCAGUAAGAACAGAGUAUUUAAUUAAAUAUUACACAAGGAUAUAAAAGCCCAGGGGAAUUGCAGU
NC_045712.1/1905206-1905087     GGUUUUCUAAAAUUAGUGUUCCCUAGUACAUAAACCAGUAAGAACAGAGUAUUUAAUUAAAUAUUACACAAGGAUGUAAAAGCCCAGGGGACUGCUGUC
NC_000003.12/4535008-4535127    GGUUUUCUAAAAUUAGUGUUCUCUAGUACAUAAACCAGUAGGAACAGAGUAUUUAAUUAAAUAUUAUACAAGGAUAUAAAAGCCCAGGGGAAUGCAGUC
NC_086015.1/8578823-8578942     GGUUUUCUAAAAUUAGUGUUCUCUAGUACAUAAACCAGUAGGAACAGAGUAUUUAAUUAAAUAUUAUACAAGGAUAUAAAAGCCCAGGGGAAUGCAGUC
NC_086017.1/14332345-14332464   GGUUUUCUAAAAUUAGUGUUCUCUAGUACAUAAACCAGUAGGAACAGAGUAUUUAAUUAAAUAUUAUACAAGGAUAUAAAAGCCCAGGGGAAUGCAGUC
NC_085926.1/4548547-4548666     GGUUUUCUAAAAUUAGUGUUCUCUAGUACAUAAACCAGUAGGAACAGAGUAUUUAAUUAAAUAUUAUACAAGGAUAUAAAAGCCCAGGGGAAUGCAGUC
NC_091263.1/36455948-36455829   GGUUUUCUAAAAUUAGUGUUCUCUAGUAUAUAAACCAGUAAGAACAGAGUAUUUAAUUAAAUAUUAUACAAGGAUAUAAAAGCCCAGGGGAAUGCAGUC
NC_087452.1/9988746-9988627     GGUUUUCUAAAAUUAGUGUUCCCUAGUACAUAAACCAGUAAGAACAGAGUAUUUAAUUAAAUAUUACACAAGGAUGUAAAAGCCCAGGGGACUGCAGUC
NW_021964193.1/8880789-8880670  GGUUUUCUAAAAUUAGUGUUCCCUAGUACAUAAACCAGUAAGAACAGAGUAUUUAAUUAAAUAUUACACAAGGAUGUAAAAGCCCAGGGGACUGCAGUC
NW_021703780.1/91111795-91111914 GGUUUUCUAAAAUUAGUGUGCUCUAGUACAUAAACCAGUAAGCACAGAGUAUUUAAUUAGCUAUUACACAAGGAUAUAAAAGCCCAGGGGAAUGCAGUC
NW_022098020.1/1965517-1965398  GGUUUUCUAAAAUUAGUGUGCUCUAGUACAUAAACCAGUAAGCACAGAGUAUUUAAUUAGCUAUUACACAAGGAUAUAAAAGCCCAGGGGAAUGCAGUC
NC_081319.1/11895678-11895559   GGUUUUCUAAAAUUAGUGUUCUCUAGUACAUAAACCAGUAAGCACAGAGUAUUUAAUUAACUAUUACACAAGGAUAUAAAAGCCCAGGGGAAUGCAGUC
NC_090835.1/11837981-11837862   GGUUUUCUAAAAUUAGUGUUCUCUAGUACAUAAACCAGUAAGCACAGAGUAUUUAAUUAACUAUUACACAAGGAUAUAAAAGCCCAGGGGAAUGCAGUC
NC_090305.1/95770079-95770198   GGUUUUCUAAAAUUAGUGUUCUCUAGUACAUAAACCAGUAAGCACAGAGUAUUUAAUUAACUAUUACACAAGGAUAUAAAAGCCCAGGGGAAUGCAGUC
NC_089228.1/11672385-11672266   GGUUUUCUAAAAUUAGUGUUCUCUAGUACAUAAACCAGUAAGCACAGAGUAUUUAAUUAACUAUUACACAAGGAUAUAAAAGCCCAGGGGAAUGCAGUC
NC_083722.1/101280131-101280250 GGUUUUCUAAAAUUAGUGUUCUCUAGUACAUAAACCAGUAAGCACAGAGUAUUUAAUUAACUAUUACACAAGGAUAUAAAAGCCCAGGGGAAUGCAGUC
NC_083104.1/11874583-11874464   GGUUUUCUAAAAUUAGUGUUCUCUAGUACAUAAACCAGUAAGCACAGAGUAUUUAAUUAACUAUUACACAAGGAUAUAAAAGCCCAGGGGAAUGCAGUC
NC_082670.1/98502770-98502889   GGUUUUCUAAAAUUAGUGUUCUCUAGUACAUAAACCAGUAAGCACAGAGUAUUUAAUUAACUAUUACACAAGGAUAUAAAAGCCCAGGGGAAUGCAGUC
NC_082692.2/92409204-92409323   GGUUUUCUAAAAUUAGUGUUCUCUAGUACAUAAACCAGUAAGCACAGAGUAUUUAAUUAACUAUUACACAAGGAUAUAAAAGCCCAGGGGAAUGCAGUC
NC_045795.1/92344359-92344478   GGUUUUCUAAAAUUAGUGUUCUCUAGUACAUAAACCAGUAAGCACAGAGUAUUUAAUUAACUAUUACACAAGGAUAUAAAAGCCCAGGGGAAUGCAGUC
NC_045773.1/11574106-11573987   GGUUUUCUAAAAUUAGUGUUCUCUAGUACAUAAACCAGUAAGCACAGAGUAUUUAAUUAACUAUUACACAAGGAUAUAAAAGCCCAGGGGAAUGCAGUC
NC_083324.2/11702385-11702266   GGUUUUCUAAAAUUAGUGUUCUCUAGUACAUAAACCAGUAAGCACAGAGUAUUUAAUUAACUAUUACACAAGGAUAUAAAAGCCCAGGGGAAUGCAGUC
NW_020173397.1/2365110-2364991  GGUUUUCUAAAAUUAGUGUUCUCUAGUACAUAAACCAGUAAGCACAGAGUAUUUAAUUAACUAUUACACAAGGAUAUAAAAGCCCAGGGGAAUGCAGUC
NC_080073.1/12045655-12045536   GGUUUUCUAAAAUUAGUGUUCUCUAGUACAUAAACCAGUAAGCACAGAGUAUUUAAUUAACUAUUACACAAGGAUAUAAAAGCCCAGGGGAAUGCAGUC
NC_041231.1/15665165-15665046   GGUUUUCUAAAAUUAGUGUUCUCUAGUACAUAAACCAGUAAGCACAGAGUAUUUAAUUAACUAUUACACAAGGAUAUAAAAGCCCAGGGGAAUGCAGUC
NC_047043.1/91263025-91263144   GGUUUUCUAAAAUUAGUGUUCUCUAGUACAUAAACCAGUAAGCACAGAGUAUUUAAUUAACUAUUACACAAGGAUAUAAAAGCCCAGGGGAAUGCAGUC
NC_064568.1/11695357-11695238   GGUUUUCUAAAAUUAGUGUUCUCUAGUACAUAAACCAGUAAGCACAGAGUAUUUAAUUAACUAUUACACAAGGAUAUAAAAGCCCAGGGGAAUGCAGUC
NW_006779955.1/2091339-2091220  GGUUUUCUAAAAUUAGUGUUCUCUAGUACAUAAACCAGUAAGCACAGAGUAUUUAAUUAACUAUUACACAAGGAUAUAAAAACCCAGGGGAAUGCAGUC

Outliers
NC_082649.1/95218120-95218242   AUCGGUUUUCUAAAAUUAGUGCUCUCUAGUACAUAAACCAGUAAGCACAGAGUAUUUAAUUAACUAUUACACAAGGAUAUAAAAGCCCAGGGGAAUGCAGUC
NW_020837966.1/26243372-26243465 UACAUAAACCAGUAAGCACA---------------------------G--AGUAUUUAAUUAACUAUUACACAAGGAUAUAAAAGCCCAGGGGAAUGCAGUC
NC_090699.1/54764490-54764600   CAACCAAAAUUAAAUGCCUCAG-UACUG-----------UGUUCUGAGACUACUUUUUUUAAUCAGUGGCACGGUCUGACAAAAUCUCA--GGAGGCAAUCU
```

NC_073643.1 - *Bubalus kerabau* - Carabao
NC_059177.1 - *Bubalus bubalis* - Domestic water buffalo
NC_056072.1 - *Ovis aries* - Sheep
NC_091078.1 - *Capricornis sumatraensis* - Sumatran serow
NC_083889.1 - *Bos javanicus* - Banteng
NW_026572372.1 - *Moschus berezovskii* - Dwarf musk deer
NW_024070193.1 - *Oryx dammah* - Oryx
NC_032671.1 - *Bos indicus* - Zebu
NW_011494766.1 - *Bison bison bison* - Plains bison
NC_091638.1 - *Bos mutus* - Wild yak
NC_030829.1 - *Capra hircus* - Goat
NW_005816350.1 - *Pantholops hodgsonii* - Tibetan antelope
NC_037349.1 - *Bos taurus* - Cattle
NC_040097.1 - *Bos indicus* x *Bos taurus* - hybrid cattle
NC_010455.5 - *Sus scrofa* - Wild boar
NC_062544.1 - *Phacochoerus africanus* - Common warthog
NC_089252.1 - *Muntiacus reevesi* - Reeves's muntjac
NC_083704.1 - *Dama dama* - European fallow deer
NC_069699.1 - *Odocoileus virginianus* - White-tailed deer
NW_012134636.1 - *Propithecus coquereli* - Coquerel's sifaka
NC_068910.1 - *Budorcas taxicolor* - Takin
NC_080198.1 - *Hippopotamus amphibius* - Hippopotamus
NC_057838.1 - *Cervus elaphus* - Red deer
NC_057407.1 - *Cervus canadensis* - Elk
NC_045712.1 - *Camelus ferus* - Wild Bactrian camel
NC_000003.12 - *Homo sapiens* - Human
NC_086015.1 - *Pan troglodytes* - Chimpanzee
NC_086017.1 - *Gorilla gorilla gorilla* - Western lowland gorilla
NC_085926.1 - *Pan paniscus* - Bonobo
NC_091263.1 - *Ovis canadensis* - Bighorn sheep
NC_087452.1 - *Camelus dromedarius* - Dromedary
NW_021964193.1 - *Vicugna pacos* - Alpaca
NW_021703780.1 - *Monodon monoceros* - Narwhal
NW_022098020.1 - *Delphinapterus leucas* - Beluga whale
NC_081319.1 - *Kogia breviceps* - Pygmy sperm whale
NC_090835.1 - *Eschrichtius robustus* - Gray whale
NC_090305.1 - *Pseudorca crassidens* - False killer whale
NC_089228.1 - *Phocoena phocoena* - Harbour porpoise
NC_083722.1 - *Eubalaena glacialis* - North Atlantic right whale
NC_083104.1 - *Lagenorhynchus albirostris* - White-beaked dolphin
NC_082670.1 - *Mesoplodon densirostris* - Blainville's beaked whale
NC_082692.2 - *Delphinus delphis* - Short-beaked common dolphin
NC_045795.1 - *Balaenoptera musculus* - Blue whale
NC_045773.1 - *Phocoena sinus* - Vaquita
NC_083324.2 - *Globicephala melas* - Long-finned pilot whale
NW_020173397.1 - *Neophocaena asiaeorientalis* - Narrow-ridged finless porpoise
NC_080073.1 - *Balaenoptera acutorostrata* - Common minke whale
NC_041231.1 - *Physeter catodon* - Sperm whale
NC_047043.1 - *Tursiops truncates* - Common bottlenose dolphin
NC_064568.1 - *Orcinus orca* - Orca
NW_006779955.1 - *Lipotes vexillifer* - Baiji

Outliers
NC_082649.1 - *Balaenoptera ricei* - Rice's whale
NW_020837966.1 - *Lagenorhynchus obliquidens* - Pacific white-sided dolphin
NC_090699.1 - *Lepisosteus oculatus* - Spotted gar (Fish)

Supplementary Fig. 14 (two previous pages) │ Alignment of mammalian sequences with similarity to bacterial guanidine-I riboswitch aptamers. Red letters identify nucleotides that match the conserved nucleotides from bacterial guanidine-I riboswitch aptamers[2]. Regions mimicking the base-paired regions P1 and P2 are shaded green and blue, respectively. Asterisks identify apparent conserved nucleotide positions that in bacterial aptamers make direct contacts with the guanidine ligand. Sequences were used to generate the consensus sequence and structural model depicted in Fig. 5c.

Supplementary Table 1. Synthetic Oligonucleotides used in this study. DNAs are depicted from 5′ to 3′.

Name	Sequence	Annotation
KK110_LA_GuanidineM-inlineF	TAATACGACTCACTATAggAGACCGAAAGCTGGGGACGACCCCAGAAAGACCAAAAGCTGGGGACGACCCCAGAAAGACCGA	Forward primer to generate template from Elephant with downstream gene *CA8* used to generate RNAs for in-line probing
KK111_LA_GdineM-inlineR	TCGGTCTTTCTGGGGTCGTCCCCAGCTTTTGGTCTTTCTGGGGTCGTCCCCAGCTTTCGGTCTccTATAGTGAGTCGTATTA	Reverse primer to generate template from Elephant with downstream gene *CA8* used to generate RNAs for in-line probing
KK112_LA_GdineM-inlineF_M1	TAATACGACTCACTATAggAGACCGAAAGCTGGGGATGACCCCAGAAAGACCAAAAGCTGGGGACGACCCCAGAAAGACCGA	Forward primer to generate mutant version of the template from Elephant with downstream gene *CA8* used to generate M1 RNAs for in-line probing
KK113_LA_GdineM-inlineR_M1	TCGGTCTTTCTGGGGTCGTCCCCAGCTTTTGGTCTTTCTGGGGTCATCCCCAGCTTTCGGTCTccTATAGTGAGTCGTATTA	Reverse primer to generate mutant version of the template from Elephant with downstream gene *CA8* used to generate M1 RNAs for in-line probing
dolphin-2F	TAATACGACTCACTATAggAGTGCCGAGCCCACAGGACGGCCTGTGGAAGCGTCAGCTGAGCCCACAGGACGGCCTGTGTGAGCGTCAG	Forward primer to generate template from dolphin with downstream gene *CACNA1C* used to generate RNAs for in-line probing
dolphin-2R	CTGACGCTCACACAGGCCGTCCTGTGGGCTCAGCTGACGCTTCCACAGGCCGTCCTG	Reverse primer to generate template from dolphin with downstream gene

	TGGGCTCGGCACTCCTATAGTGAGTCGTATTA	*CACNA1C* used to generate RNAs for in-line probing
KK123_LA_GdineM_tandem2-WT_F	TAATACGACTCACTATAggAAGCTGGGGATGACCCCAGAAAGACCGAAAGCTGGGGACGACCCCAGAAAGACCGAAAGCTGGGGACGACCCCAGAAAGACCGAAAGCTGGGGACGACCCCAGAAAGACCGA	Forward primer to generate template from Elephant-second tandem with downstream gene *CA8* used to generate RNAs for in-line probing
KK124_LA_GdineM_tandem2-WT_R	TCGGTCTTTCTGGGGTCGTCCCCAGCTTTCGGTCTTTCTGGGGTCGTCCCCAGCTTTCGGTCTTTCTGGGGTCGTCCCCAGCTTTCGGTCTTTCTGGGGTCATCCCCAGCTTccTATAGTGAGTCGTATTA	Reverse primer to generate template from Elephant-second tandem with downstream gene *CA8* used to generate RNAs for in-line probing
KK125_LA_GdineM_tandem2- M1_R	TCGGTCTTTCTGGGGTCGTCCCCAGCTTTCGGTCTTTCTGGGGTCGTCCCCAGCTTTCGGTCTTTCTGGGGTCATCCCCAGCTTTCGGTCTTTCTGGGGTCATCCCCAGCTTccTATAGTGAGTCGTATTA	Reverse primer to generate template from Elephant-second tandem M1 with downstream gene *CA8* used to generate RNAs for in-line probing
KK129_GuaI_HumanLong_WT_gblock	TAATACGACTCACTATAGGGTTAGAAAGAACCAGCTAATCGGTTTTCTAAAATTAGTGTTCTCTAGTACATAAACCAGTAGGAACAGAGTATTTAATTAAATATTATACAAGGATATAAAAGCCCAGGGGAATGCAGTC	IDT gBlock (dsDNA) for in-line probing
KK131_GuaI_HumanMutlong_F	TAATACGACTCACTATAGGGTTAGAAAGAACCAGCTAATCGGTTTTCTAAAATTAGTGTTCTCTAGTACATAAACCAGTAGGAACAGAGTATTTAATTAAATATTATACAAGGATATAAAAGCCCAGAGAAATGCAGTC	Forward primer to generate mutant template from human guanidine-I with downstream gene used to generate RNAs for in-line probing

KK132_ GuaI_Human Mutlong_R	GACTGCATTTCTCTGGGCTTTATATCCTT GTATAATATTTAATTAAATACTCTGTTCCTA CTGGTTTATGTACTAGAGAACACTAATTTT AGAAAACCGATTAGCTGGTTCTTTCTAAC CCTATAGTGAGTCGTATTA	Reverse primer to generate mutant template from human guanidine-I with downstream gene used to generate RNAs for in-line probing
KK135_ PISD_RA2L _F	TAATACGACTCACTATAggCACCACATCAC CTCAGGGACGGCCCTGAGCTCACACCAC ATCACCTCAGGGACGGCCCTGAGCTCAC ACC	Forward primer to generate guanidine-II WT template from bird with downstream gene *PISD* used to generate RNAs for in-line probing
KK136_ PISD_RA2L _R	GGTGTGAGCTCAGGGCCGTCCCTGAGGT GATGTGGTGTGAGCTCAGGGCCGTCCCTG AGGTGATGTGGTGccTATAGTGAGTCGTAT TA	Reverse primer to generate guanidine-II WT template from bird with downstream gene *PISD* used to generate RNAs for in-line probing
**For genetics**		
CA8 gblock	CCTTACCGCATTGAAGGCCAAAAAACTGC TGCCTTCGGATCCTTTACGACAAATTGCA AAAATAATGTTGTCCTTTTAAATAAGATCT GATAAAATGTGAACTAATTTCATAGAAGG AGGAAATGGCTGATCTTAGTTTCATCGAA GATACCGTGGCGTTTCCTGAGAAGGAAG AGGACGAAGAGGAAGAAGAGGAAGGGG TGGAATGGGGGTACGAAGAGGGCGTTGA GTGGGGATTGGTTTTTCCGGACGCCAATG GTGAATATCAGTCTCCAATTAACCTGAAC AGCCGCGAAGCCAGATATGACCCTTCCCT GCTGGATGTCCGCCTCTCTCCTAATTATGT AGTCTGCAGAGACTGTGAGGTTACTAATG ATGGACACACTATCCAGGTCATATTAAAA AGTAAATCCGTCCTCAGTGGCGGGCCGTT	GenScript gBlock (dsDNA) containing *LysC* promoter with *EcoRI* and *BamHI* restriction site sequences for cloning in pDG1664 plasmid.

	GCCGCAAGGGCATGAATTTGAACTTTACG AAGTGCGGTTTCATTGGGGTCGCGAAAA CCAGCGCGGTTCAGAGCATACCGTGAACT TTAAGGCTTTCCCGATGGAACTCCACTTG ATACATTGGAATAGCACGCTCTTCGGCAG CATAGATGAGGCCGTAGGAAAACCGCATG GCATAGCTATCATCGCCTTGTTCGTACAGA TTGGCAAGGAACACGTAGGGCTGAAAGC GGTGACTGAAATTCTTCAAGATATTCAGT ACAAGGGGAAGTCCAAAACTATTCCATGT TTTAACCCAAATACACTCCTGCCAGATCC GCTCCTGCGCGATTACTGGGTTTATGAAG GCTCTCTCACCATACCGCCGTGCAGCGAA GGTGTGACCTGGATTCTCTTCCGGTACCC GCTTACAATTTCCCAACTCCAGATCGAGG AATTTCGCCGTTTGCGTACCCATGTTAAG GGGGCAGAGTTGGTAGAGGGATGCGACG GCATTCTCGGAGATAATTTCCGGCCGACA CAACCGTTATCCGACCGCGTGATTCGGGC AGCTTTCCAATAGAAGCTTATCGAATTCG ATAACCCTAAAGTTATGGAAATAAGACTT AGAAGC	
KK117_CA8 _GdM_F	CCTTACCGCATTGAAGGC	Forward primer used for amplification and confirmation of *CA8* gene by sequencing.
KK118_CA8 _GdM_R	GCTTCTAAGTCTTATTTCCATAACTTTAGG	Reverse primer used for amplification and confirmation of *CA8* gene by sequencing.
Y218C_FP	GAGAGCCTTCACAAACCCAGTAATCGCG CAGGA	Forward primer used in site-directed mutagenesis to

		generate Y218C mutant of CA8.
Y218C_RP	TCCTGCGCGATTACTGGGTTTGTGAAGGC TCTC	Forward primer used in site-directed mutagenesis to generate Y218C mutant of CA8.
