# Supplementary figures and images for: Guanidine aptamers are present in vertebrate RNAs associated with calcium signaling and neuromuscular function
Source: Nat Commun. 2025 Aug 9;16:7362. doi: 10.1038/s41467-025-62815-6 (PMC12335538; doi:10.1038/s41467-025-62815-6)

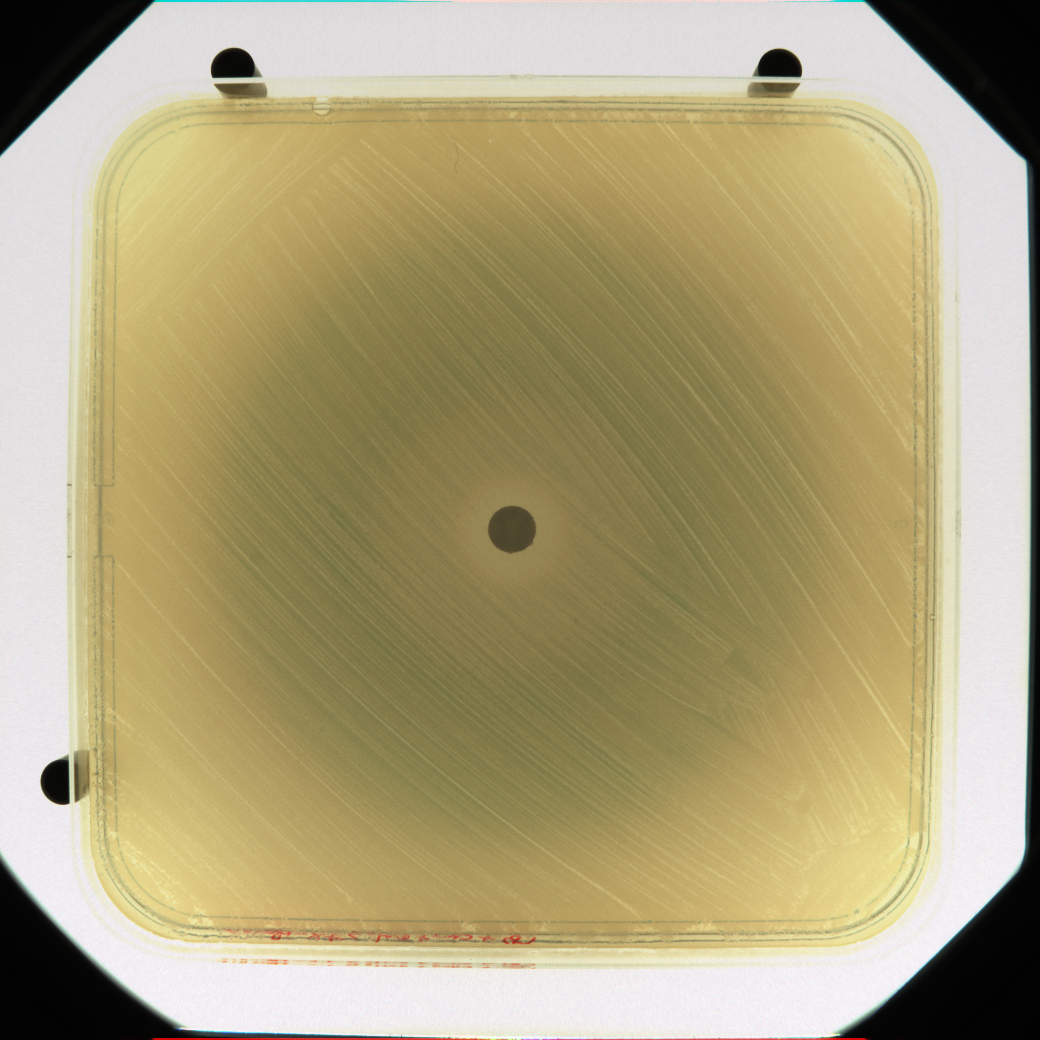

Supplement: Supplementary file 4 — Source Data [file 41467_2025_62815_MOESM4_ESM.zip › Guanidine Aptamers Raw Data_Source Data/Fig. 3e delta ykkCD CA8 Mutant Plate.tif]

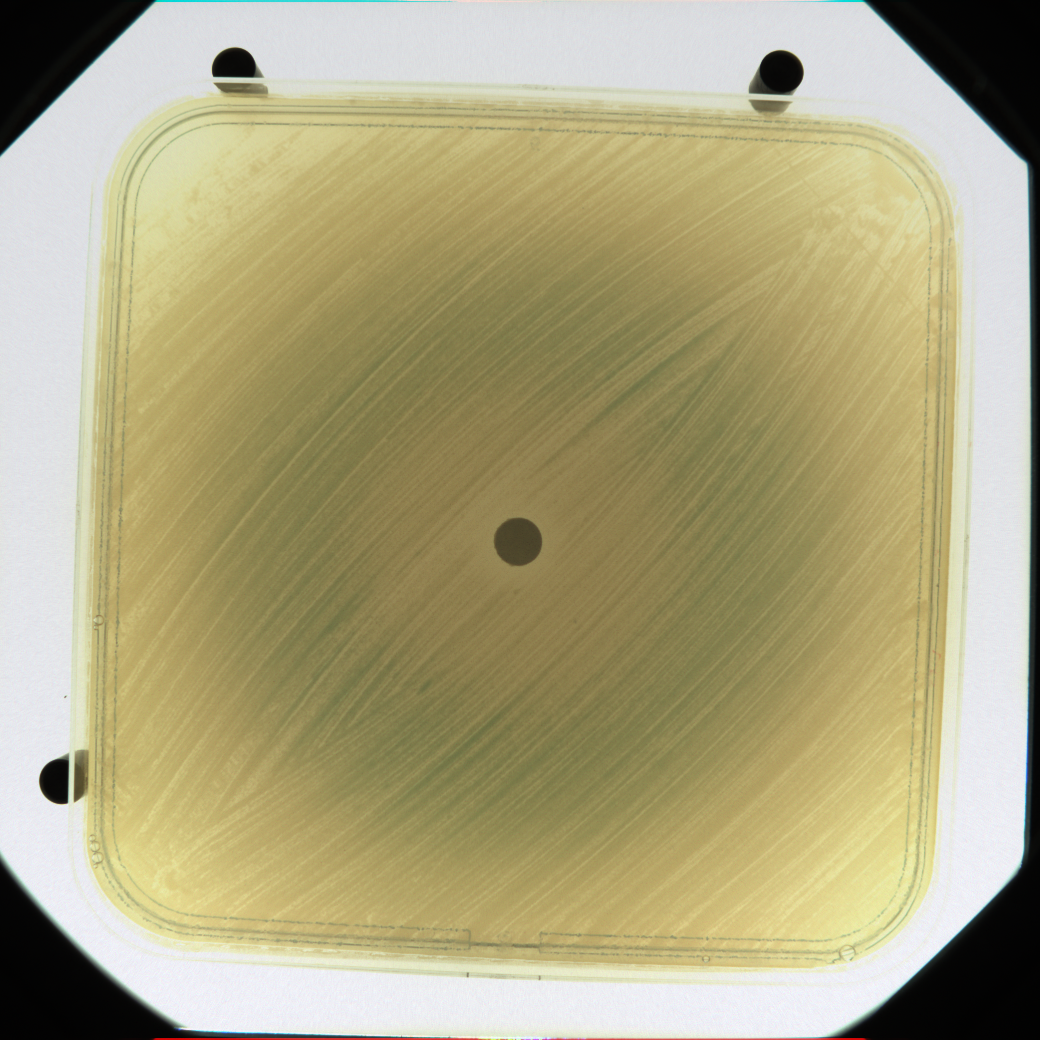

Supplement: Supplementary file 4 — Source Data [file 41467_2025_62815_MOESM4_ESM.zip › Guanidine Aptamers Raw Data_Source Data/Fig. 3e delta ykkCD CA8 Plate.tif]

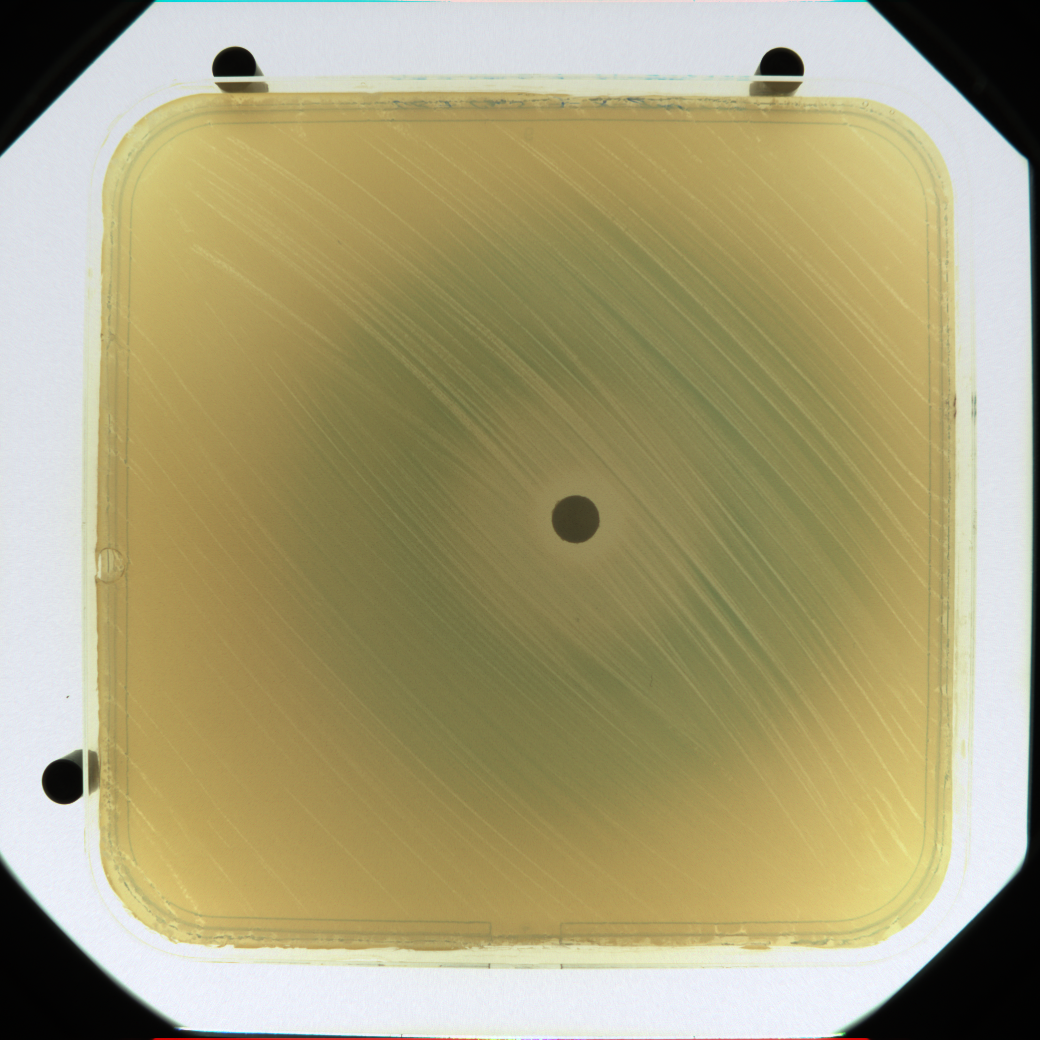

Supplement: Supplementary file 4 — Source Data [file 41467_2025_62815_MOESM4_ESM.zip › Guanidine Aptamers Raw Data_Source Data/Fig. 3e delta ykkCD No Insert Plate.tif]

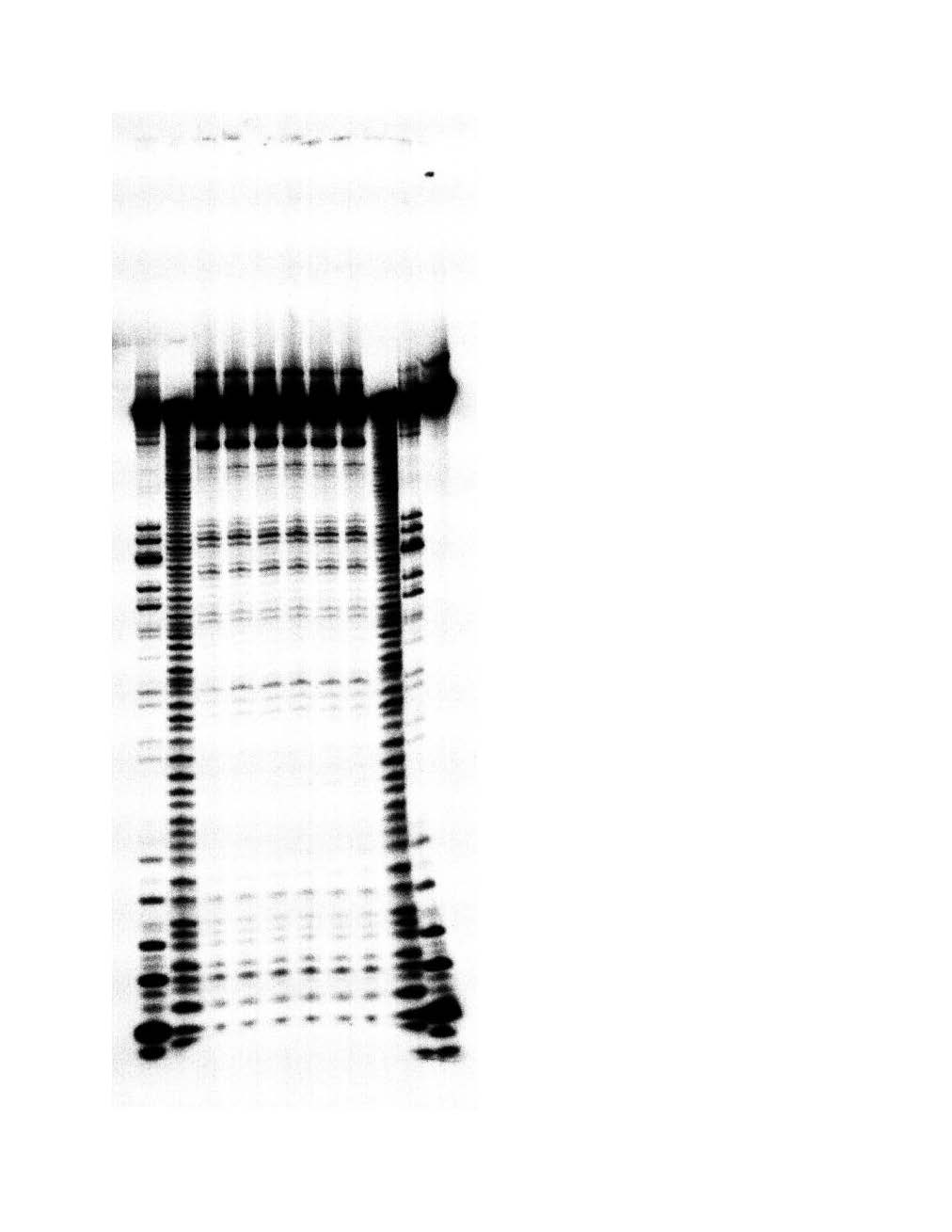

Supplement: Supplementary file 4 — Source Data [file 41467_2025_62815_MOESM4_ESM.zip › Guanidine Aptamers Raw Data_Source Data/Original Gel Image for Supplementary Fig. 12b.jpg]

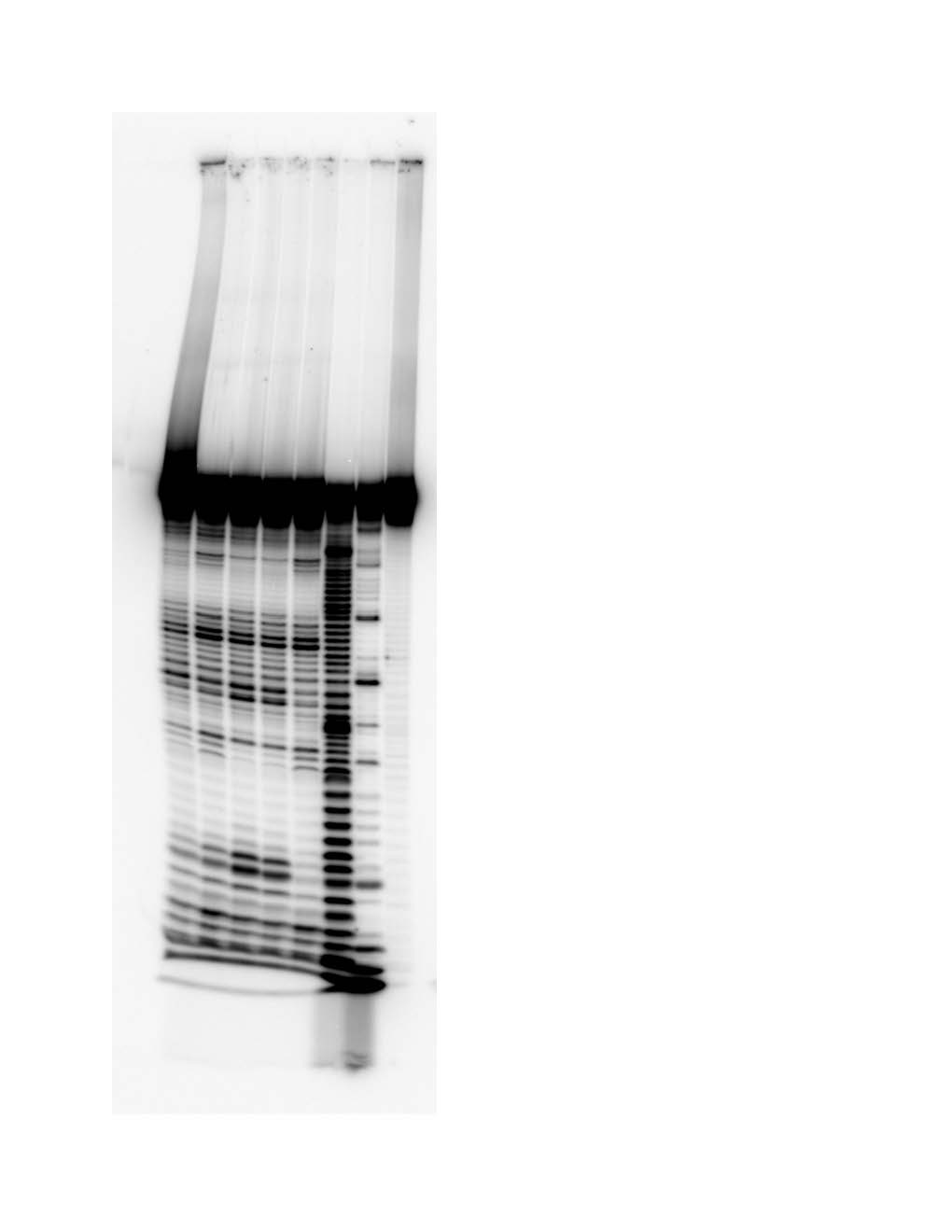

Supplement: Supplementary file 4 — Source Data [file 41467_2025_62815_MOESM4_ESM.zip › Guanidine Aptamers Raw Data_Source Data/Original Gel Image for Supplementary Fig. 4c Reflected.jpg]
